# Current landscape of innovative drug development and regulatory support in China

**DOI:** 10.1038/s41392-025-02267-y

**Published:** 2025-07-22

**Authors:** Ruirong Tan, Hua Hua, Siyuan Zhou, Zhimin Yang, Changming Yang, Guo Huang, Jin Zeng, Junning Zhao

**Affiliations:** 1 Key Lab.: Biological Evaluation of TCM Quality of the State Administration of Traditional Chinese Medicine, Translational Chinese Medicine Key Laboratory of Sichuan Province, Sichuan Institute for Translational Chinese Medicine, Sichuan Academy of Chinese Medicine Sciences, Chengdu, China; 2 Center for Drug Evaluation, National Medical Products Administration (NMPA), Beijing, China; 3 National Medical Products Administration (NMPA), Beijing, China; 4 National Center for Nanoscience and Technology, Beijing, China

**Keywords:** Drug regulation, Drug regulation

## Abstract

The global pharmaceutical landscape remains dynamic and competitive, shaped by advancements in first-in-class therapies and breakthrough technologies. The United States has maintained its leadership in first-in-class therapies and breakthrough technologies, driven by advanced regulatory pathways, significant multinational corporation investments, a robust Research and Development (R&D) workforce, and continuous technological innovation. Additionally, global impact of the Food and Drug Administration (FDA) is further amplified through collaborations like Project Orbis, which facilitates simultaneous reviews of cancer treatments by multiple regulatory authorities worldwide. Europe, while historically strong, faces growing challenges in maintaining its competitive edge, particularly due to protracted regulatory timelines and complex coordination among its member states. In this competitive global environment, China has rapidly transformed from a generics-dominated market to a key player in innovative drug development. This article reviews China’s progress in innovative drug R&D from 2019 to 2023, emphasizing regulatory modernization, clinical trial advancements, and the emergence of novel therapies. By comparing China’s developments with above global counterparts, this review highlights the country’s achievements in regulatory efficiency, clinical trial progress, and the development of innovative therapies such as biologics and cell and gene therapies. Through this comparative analysis, the article underscores how China’s evolving policy-driven innovation ecosystem has positioned it as a growing leader in global drug development. The review examines how enhanced regulatory efficiency, clinical trial progress, manufacturing capabilities, and international collaboration have bolstered China’s growing influence, while also discussing the future opportunities and challenges it faces in shaping global pharmaceutical innovation and development.

## Introduction

Innovative drug development has become a central focus of the global pharmaceutical industry, driven by advancements in biomedical technologies, regulatory innovations, and unmet medical needs. Across major global markets, such as the United States and Europe, significant advancements have been achieved in diverse areas of pharmaceutical innovation, encompassing novel therapeutic platforms, breakthroughs in precision medicine, and the integration of cutting-edge technologies, such as artificial intelligence, as well as the application of real-world evidence in drug development.^1–7^ Leading regulatory agencies like the U.S. FDA and European Medicines Agency (EMA) have implemented innovative approaches, such as expedited approval pathways, to facilitate the development and commercialization of these therapies.^8–13^ Additionally, both agencies have expanded their global influence through collaborations like Project Orbis,^14^ which allows simultaneous reviews of cancer treatments by multiple regulatory bodies,^15–19^ and the establishment of the African Medicines Agency (AMA),^20^ based on the EMA’s model, which enhances regulatory capacity across Africa.^21–25^ Simultaneously, emerging markets, such as China, have intensified their efforts to compete on a global scale, aligning regulatory frameworks with international standards and focusing on advancements in innovative pharmacological developments.

In parallel with global advancements, China has emerged as a pivotal player in the pharmaceutical industry, transitioning from a generics-focused market to one increasingly driven by innovation.^26–29^ The evolution of China’s regulatory framework has been pivotal in facilitating this progress. Beginning with the establishment of the State Drug Administration (SDA) in 1998^30,31^ and its transformation into the National Medical Products Administration (NMPA) in 2018,^31,32^ China has progressively modernized its regulatory system. Guided by principles of scientific rigor and efficiency, NMAP has supported the China’s shift towards a more innovation-driven pharmaceutical industry. Major regulatory changes has been implemented to align with international standards, including streamlining its drug approval pathways and adopting International Council for Harmonisation (ICH) guidelines.^1,33–37^ The NMPA has been central to the efforts and initiatives such as the “Major New Drug Development” National Science and Technology Project and the “Drug Regulatory Science Action Plan” which have provided strong support for innovative drug research and development.^38,39^ These measures have not only accelerated the review and approval of new drugs but also fostered the development of advanced therapeutic approaches, such as cell and gene therapies, further integrating China into the global pharmaceutical ecosystem.^40–43^

China’s progress in innovative drug development has been further driven by a combination of increased R&D investment, advancements in clinical trial efficiency, and the emergence of globally competitive therapies. Between 2019 and 2023, China’s pharmaceutical industry witnessed a significant rise in Investigational New Drug (IND) applications and New Drug Applications (NDA), reflecting a rapidly growing pipeline of innovative therapies.^44,45^ Notably, the acceleration of clinical trial approvals and the increasing integration of China into global multicenter studies underscore the country’s expanding role in global pharmaceutical innovation.^30,45^ This progress is complemented by a sharp increase in the number of first-in-class drugs, next-generation biologics, and novel technologies like cell and gene therapies.^40,41^ These achievements highlight China’s dual focus on addressing domestic public health challenges while significantly enhancing its role in global pharmaceutical innovation.

Despite China’s remarkable advancements, the global landscape of innovative drug development remains highly dynamic and competitive, with regions such as the United States and Europe continuing to lead in several key areas. The United States, driven by well-established regulatory frameworks such as the FDA’s Breakthrough Therapy Designation and Accelerated Approval pathways, continues to excel in the development of innovative therapies.^8,11^ Europe continues to be a global hub for clinical research, and the EMA has made significant strides in harmonizing regulatory frameworks across member states, facilitating multi-country trials and promoting innovation.^2,46–48^ However, the region faces challenges such as extended approval timelines and intricate administrative requirements, which could impact its ability to remain competitive in the rapidly advancing global pharmaceutical sector. In comparison, China’s strengths lie in its rapidly increasing clinical trial efficiency, policy-driven innovation ecosystem, and significant advancements in biopharmaceutical manufacturing capabilities. This evolving dynamic highlights the global interconnectivity of pharmaceutical R&D and the need for continued international collaboration to address unmet medical needs and advance drug innovation.

Looking ahead, the continued evolution of China’s innovative drug landscape will depend on its ability to sustain global competitiveness while addressing key challenges, such as regulatory alignment, investment in R&D, and enhancing clinical trial efficiency. Strengthening alignment with international regulatory standards and fostering deeper collaboration with global pharmaceutical companies can further enhance China’s integration into the global ecosystem. Meanwhile, the country’s focus on emerging technologies positions it as a critical player in addressing global health priorities. By leveraging its growing expertise in clinical trial efficiency, innovative drug manufacturing, and policy-driven support, China has the potential to contribute significantly to the development of life-saving therapies worldwide, particularly in areas such as cell-based therapies, exemplified by CAR-T therapy for cancer treatment, and gene therapy, where advancements in treating genetic disorders like thalassemia and hemophilia have garnered global attention.

This review systematically examines China’s progress in innovative drug development and regulation from 2019 to 2023, placing it within a global context. The article explores key aspects such as Investigational New Drug (IND) and New Drug Application (NDA) processes, advances in clinical trials, and evaluations of cutting-edge technologies and therapies. Additionally, it provides an international comparison of regulatory efficiency, investment trends, and scientific innovation, with a focus on the United States and European Union. This review highlights China’s achievements and challenges within the global context, offering insights into the evolving pharmaceutical landscape. It serves as a resource for policymakers, researchers, and industry leaders aiming to advance innovation and address global health needs.

## Current status of innovative drug development and approval in China within a global context

### Definition and classification of innovative drugs

According to the “Drug Administration Law of the People’s Republic of China,” the “drugs” include TCM, chemical drugs, and biologics.^49^ In August 2015, the State Council issued the “Opinions on Reforming the Review and Approval System for Drugs and Medical Devices,” which categorized drugs into innovative drugs and generic drugs.^50^ In parallel, similar reforms have been implemented for medical devices, including the introduction of the “Special Approval Procedure for Innovative Medical Devices,” which accelerates the approval process for breakthrough devices with significant clinical value, further supporting innovation in this sector.

The definition of innovative drugs has been modified from the erstwhile criterion of “drugs not previously introduced to the Chinese market” to “drugs not yet introduced to the global market”. This alteration has broadened the scope of Chinese innovative drugs from being “novel to China” to being “novel to the world.”^50^ To encourage new drug development, ensure rigorous review and approval processes, enhance drug quality, and promote industrial upgrades, the NMPA has proactively improved the drug registration classification system. Chemical drugs are now stratified into five categories, preventive or therapeutic biologics into three classes, and TCM into four classes. Category 1 drugs in each classification represent innovative drugs.^51–54^ As illustrated in Table 1, the precise definitions and classifications of Category 1 Innovative Drugs for TCM, chemical drugs, and biologics are delineated. Unless explicitly stated otherwise, the term “innovative drugs” in this paper typically denotes drugs recognized as Category 1 under the prevailing “Drug Registration Administrative Measures,” including TCM, chemical drugs, and biologics, distinguishing them from new drugs, which also include modified new drugs and other types.

**Table 1:** Definitions and classifications of Category 1 innovative drugs in China

Innovative drugs	Definition	Subcategories
Category 1 chemical drugs	Innovative drugs that have not been marketed domestically or internationally. These drugs contain new, structurally defined compounds with pharmacological activity and clinical value.^16^	
Category 1 preventive biological products (vaccines)	Vaccines that have not been marketed domestically or internationally.^17^	1.1 Vaccines for diseases with no effective preventive measures. 1.2 Vaccines developed from new antigen forms based on existing marketed vaccines. 1.3 Vaccines containing new adjuvants or novel adjuvant systems. 1.4 Multivalent or combined vaccines containing new antigens or new antigenic forms.^17^
Category 1 therapeutic biological products	Therapeutic biological products that have not been marketed domestically or internationally.^17^	
Category 1 traditional Chinese medicines (TCMs)	New TCM formulations not included in the National Drug Standards, Drug Registration Standards, or the “Ancient Classical Formula Directory” issued by national TCM authorities. These formulations must have clinical value and not be marketed internationally.^18^	1.1 TCM compound formulations. 1.2 Extracts and their preparations derived from single substances such as plants, animals, or minerals. 1.3 New medicinal materials and their preparations.^18^

Expanding on this framework, it is important to compare China’s classification of innovative drugs with the standards established by international regulatory authorities such as the United States FDA and the EMA. In the U.S., according to the standards set by the FDA, innovative drugs encompass both New Molecular Entities (NMEs) and new products approved through a Biologics License Application (BLA).^55–61^ NMEs refer to chemical or biological drugs that contain an active moiety never before approved by the FDA, emphasizing the novelty of the molecular structure or mechanism of action.^62–66^ These drugs are evaluated under the New Drug Application (NDA) pathway. In addition to NMEs, biologics approved through BLAs, including monoclonal antibodies, therapeutic proteins, vaccines, gene therapies, and cell-based therapies, are also recognized as innovative drugs.^67–71^ These biologics embody groundbreaking advancements in therapeutic development, offering targeted and highly specific mechanisms of action to address complex and unmet medical needs. By leveraging the intricacies of biological systems, biologics have revolutionized treatments for cancers, autoimmune diseases, genetic disorders, and infectious diseases, often providing solutions where traditional small-molecule drugs have limited efficacy.^72–78^ Both NMEs and BLAs undergo rigorous FDA evaluations to ensure safety, efficacy, and manufacturing consistency. This dual framework reflects the FDA’s commitment to fostering innovation across a wide spectrum of pharmaceutical and biological products, providing a regulatory foundation for advancing treatments for unmet medical needs.

The EMA defines an innovative medicine as “a medicine that contains an active substance or combination of active substances that has not been authorized before.”^79^ This definition emphasizes the novelty of the active components in the therapeutic product.^80–85^ The EMA does not have a specific classification system for innovative drugs like the FDA’s NMEs. Instead, innovation is assessed through the potential therapeutic benefits, addressing unmet medical needs, and the clinical significance of the product. Drugs and therapies that meet these criteria can access EMA programs such as Priority Medicines Scheme (PRIME)^86–90^ and Accelerated Assessment,^91–94^ which are designed to support the development and authorization of innovative treatments. These efforts aim to foster the development of new treatments that address unmet medical needs within the European Union.

A key distinction between China’s regulatory framework and that of the FDA and EMA lies in their respective priorities and approaches to innovative drug classification. The NMPA’s system aims to align with international standards while simultaneously fostering domestic innovation by providing incentives for local entities to develop novel drugs that can compete globally. This dual emphasis ensures the integration of Chinese innovation into the global pharmaceutical landscape while fostering local research and development. In contrast, both the FDA and EMA adopt classification systems that focus on therapeutic and molecular uniqueness, independent of the drug’s origin. For instance, the FDA’s NMEs and EMA’s innovative drug evaluation system focus on the drug’s novelty and clinical benefits.

Another significant difference lies in the treatment of TCM. China’s framework explicitly integrates TCM within its classification of innovative drugs, recognizing its cultural and industrial significance. This approach is unique to China and highlights a region-specific priority not shared by Western systems. Neither the FDA nor EMA includes traditional or herbal medicines in their primary innovation frameworks, as their focus remains on molecularly defined and evidence-based therapeutic products. These differences underscore China’s efforts to balance its domestic priorities with international competitiveness, while the FDA and EMA adopt a more universal and origin-neutral approach to defining and regulating innovative drugs.

### IND applications and approvals for innovative drugs: China’s role in the global landscape

**Fig. 1:**
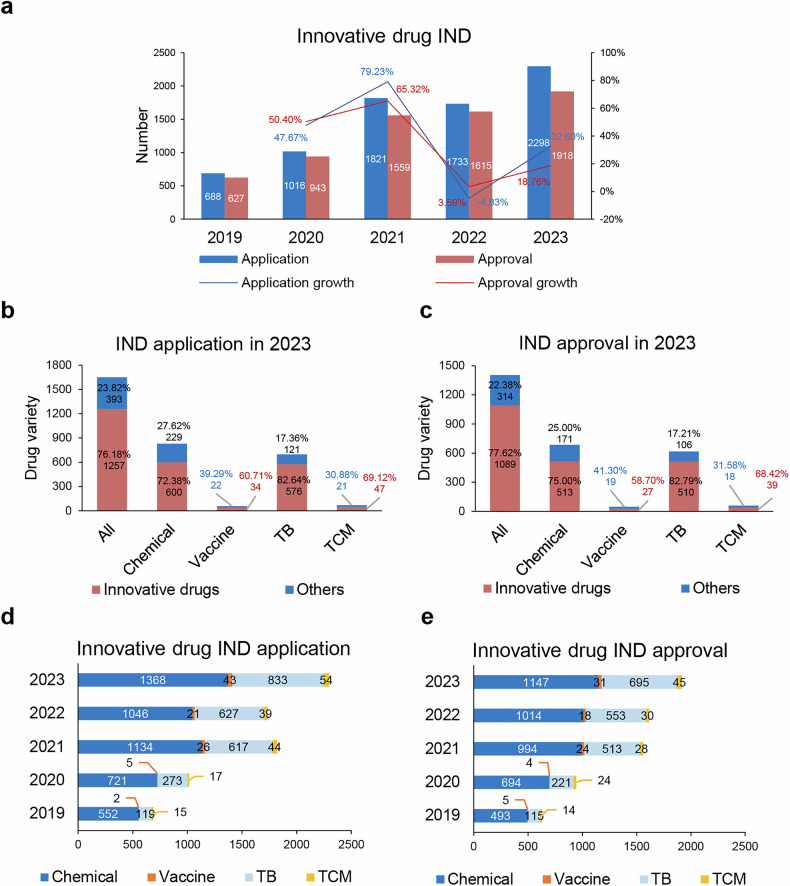
Overview of IND applications and approvals for innovative drugs in China from 2019 to 2023. **a** Number of innovative drug IND applications and approvals from 2019 to 2023, along with their respective growth rates. **b** Number and proportion of innovative drug varieties within the total drug varieties in IND applications of 2023. The groups are classified as: all drugs, chemical drugs, preventive biologics (vaccine), therapeutic biologics (TB), and TCM. **c** Number and proportion of innovative drug varieties within the total drug varieties in IND approvals of 2023. **d** Number of chemical drug, vaccine, TB and TCM among innovative drug IND applications from 2019 to 2023. **e** Number of chemical drug, vaccine, TB, and TCM among approved innovative drug INDs from 2019 to 2023. Data for this figure were derived from 2019 - 2023 Annual Drug Evaluation Reports published by CDE, NMPA.^95–99^

As depicted in Fig. 1a, the number of INDs registration applications of innovative drug accepted by the NMPA has increased annually over the last five years.^95–99^ The count of accepted applications from 688 in 2019 surged to 2298 in 2023, marking a compound annual growth rate (CAGR, calculated by dividing the ending value by the beginning value, then taking the result to the power of the reciprocal of the number of years, and finally subtracting 1) of 35.19% (Fig. 1a). Similarly, the number of approvals of innovative drug IND applications also increased from 627 in 2019 to 1918 in 2023, with a CAGR of 32.25% (Fig. 1a). In 2023, the NMPA accepted a total of 2997 new drug IND applications, covering 1650 products. Among these, 2298 applications were for innovative drugs, corresponding to 1257 products, which accounted for 76.18% of the total 1650 products (Fig. 1b). In 2023, the NMPA also approved for 2461 IND applications (covering 1403 products), with 1918 being innovative drug INDs (covering 1089 products), accounting for 77.62% of the total IND product count (Fig. 1c).

Regarding the development of innovative chemical drugs, from 2019 to 2023, a total of 4821 innovative chemical drug IND applications were accepted (552; 721; 1134; 1,046; and 1368 per annum, respectively) (Fig. 1d). In 2023, 1368 innovative chemical drug IND applications (covering 600 products) were accepted, representing a 30.78% year-on-year increase compared to the previous year, constituting 72.38% of all chemical drug IND products that year (Fig. 1b). From 2019 to 2023, 4342 innovative chemical drug INDs were approved/suggested for approval (493; 694; 994; 1014; and 1147 per annum, respectively) (Fig. 1e). In 2023, 1147 innovative chemical drug INDs (513 products) were approved/suggested for approval, representing a year-on-year increase of 13.12% compared to the previous year, accounting for 75% of all approved chemical drug IND products that year (Fig. 1c).

Regarding innovative preventive biologics (vaccine), from 2019 to 2023, 95 innovative preventive biologics IND applications were accepted (2; 5; 26; 21; and 43 per annum, respectively) (Fig. 1d). In 2023, 43 innovative preventive biologics IND applications (34 products) were accepted, representing a year-on-year increase of 104.76% compared to the previous year, accounting for 60.71% of all preventive biologics IND products that year (Fig. 1b). From 2019 to 2023, 89 innovative preventive biologics INDs were approved/suggested for approval (5; 4; 24; 18; and 31 per annum, respectively) (Fig. 1e). In 2023, 31 innovative preventive biologics INDs (27 products) were approved/suggested for approval, representing a year-on-year increase of 72.22% compared to the previous year, accounting for 58.70% of all approved preventive biologics IND products that year (Fig. 1c).

Regarding innovative therapeutic biologics, from 2019 to 2023, 2350 innovative therapeutic biologics IND applications were accepted (119; 273; 617; 627; and 833 per annum, respectively) (Fig. 1d). In 2023, 833 innovative therapeutic biologics IND applications (576 products) were accepted, representing a year-on-year increase of 32.85% compared to the previous year, accounting for 82.64% of all therapeutic biologics IND products that year (Fig. 1b). From 2019 to 2023, 1761 innovative therapeutic biologics INDs were approved/suggested for approval (115; 221; 513; 553; and 695 per year, respectively) (Fig. 1e). In 2023, 695 innovative therapeutic biologics INDs (510 products) were approved/suggested for approval, representing a year-on-year increase of 25.68% compared to the previous year, accounting for 82.79% of all approved therapeutic biologics IND products in 2023 that year (Fig. 1c).

Regarding innovative TCMs, from 2019 to 2023, 154 innovative TCM IND applications were accepted (15; 17; 44; 39; and 54 per year, respectively) (Fig. 1d). In 2023, 54 innovative TCM IND applications (47 products) were accepted, representing a year-on-year increase of 38.46% compared to the previous year, accounting for 69.12% of all TCM IND products that year (Fig. 1b). From 2019 to 2023, 127 innovative TCM INDs were approved/suggested for approval (14; 24; 28; 30; and 45 per year, respectively) (Fig. 1e). In 2023, 45 innovative TCM INDs (39 products) were approved/suggested for approval, representing a year-on-year increase of 50.00% compared to the previous year, accounting for 68.42% of all approved TCM IND products in 2023 that year (Fig. 1c).

The statistics presented above include both conventional medicines and TCMs, as TCMs play an integral role in China’s pharmaceutical innovation. However, it is important to note that TCMs are regulated separately under the NMPA and are not treated as a distinct category by the FDA or the EMA. These regulatory agencies do not specifically categorize TCMs in their drug approval processes. Therefore, the data reflects the progress of both conventional medicines and TCMs in China, with a clear distinction between the two. Therefore, when comparing statistics of China to those of its counterparts, the differing regulatory frameworks and conditions should be taken into account.

The substantial increase in the number of IND applications and approvals for innovative drugs in China reflects the country’s rapid advancements in pharmaceutical research and development. This growth demonstrates China’s emergence as a significant contributor to global drug innovation, with its IND process increasingly integrated into international clinical research efforts. The rising efficiency of IND applications and approvals, coupled with the volume of innovative drug submissions, positions China as a competitive player in the global pharmaceutical landscape.

China’s IND process has become increasingly streamlined, demonstrating a significant improvement in regulatory efficiency. In 2023, the NMPA accepted 2298 innovative drug IND applications, reflecting a substantial increase compared to previous years. Of these, 1918 INDs were approved, representing an approval-to-submission ratio exceeding 80%. This robust performance highlights China’s increasing capacity for supporting innovative drug development. When compared globally, the U.S. FDA typically receives about 1500 new IND submissions annually, with around 91% of applications allowed to proceed without clinical holds.^100–102^ Although the EMA does not have a direct IND system like the FDA or NMPA, its Clinical Trial Application (CTA) process receives significant volumes annually, with strong approval rates in line with global norms.^103–105^ The sheer volume of IND applications accepted and approved by the NMPA highlights China’s rapidly expanding pharmaceutical ecosystem.

China’s increasing IND activity has also positioned it as a competitive hub for global clinical research. According to the ClinicalTrials.gov database which includes clinical studies involving human participants conducted worldwide, China has significantly increased its presence in global clinical trials, with 57,667 trials conducted as of 2023, accounting for 12.7% of global clinical trials, compared to the United States’ 162,523 trials (35.9%).^106,107^ This marks a substantial rise for China, which had only conducted 702 trials as of 2003, representing just 3.2% of global trials at the time, compared to the United States’ 11,915 trials (54.7%).^106,107^ By 2013, the gap had begun to narrow, with China conducting 16,122 trials (9.5% of global trials), while the United States conducted 73,634 trials (43.4%).^106,107^ While the U.S. remains the dominant force, its global share has steadily declined as countries like China have emerged as key players. Unlike the U.S., which benefits from a long-established infrastructure and strong R&D investments, China’s growth has been driven by streamlined regulatory processes, government incentives, and an expanding clinical trial ecosystem.

China has surpassed Europe in clinical trial activity, reflecting its rapid growth and increasing global competitiveness. According to the European Federation of Pharmaceutical Industries and Associations (EFPIA) and IQVIA, Europe’s share of commercially sponsored clinical trials dropped from 22% in 2013 to 12% in 2023,^108–110^ reflecting a sharp decline over the past decade. The reduction may be due to fragmented regulatory frameworks following Brexit and the relocation of the EMA from the UK to Holland, which has caused disharmonization in clinical trials across European countries.^111^ Additionally, the COVID-19 pandemic has further exacerbated these challenges by disrupting trial operations, approval processes, and patient recruitment.^112^

New strategies to address the challenges brought about by the disharmonisation of clinical trials following Brexit, the relocation of the EMA, and the impact of COVID are being developed, with promising results aimed at improving the regulatory environment and ensuring more efficient trial operations in Europe.^113^ In contrast, China has significantly expanded its share of commercially sponsored clinical trials, increasing from 9% in 2018 to 18% in 2023.^108–110^ This shift is further supported by collaborative efforts between multinational pharmaceutical companies and Chinese research institutions, leveraging the country’s streamlined IND processes and expanding R&D capabilities.

### NDA applications and approvals for innovative drugs in China and international markets

**Fig. 2:**
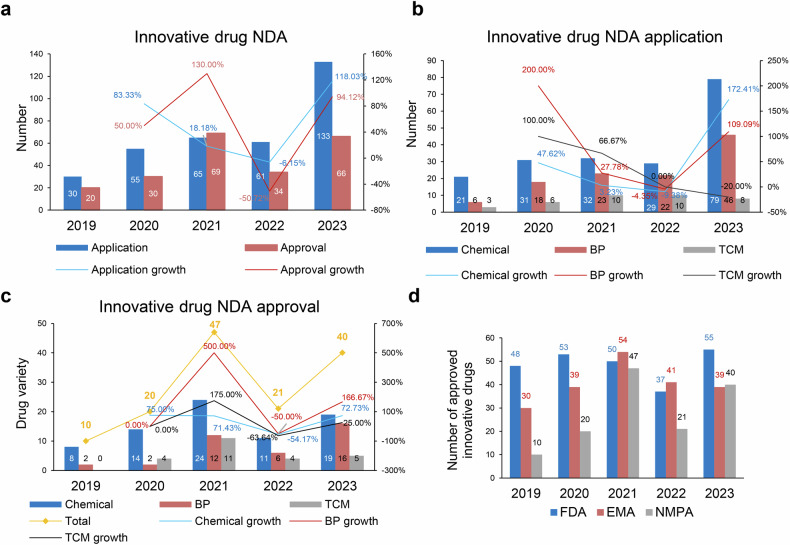
Overview of NDA applications and approvals for innovative drugs in China from 2019 to 2023. **a** Number of innovative drug NDA applications and approvals from 2019 to 2023, along with their respective growth rates. **b** Number of NDA applications for chemical drugs, biological products (BP), and TCM among innovative drug NDA applications from 2019 to 2023, along with their respective growth rates. **c** Number of drug varieties of chemical drugs, BP, and TCM among innovative drug NDAs approved from 2019 to 2023, along with their respective growth rates. The total number of approved drug varieties each year is indicated by yellow-marked symbols and numbers. **d** Number and growth rate of out-licensing deals of Chinese innovative drugs. Data for this figure were derived from 2020 - 2023 Annual Report on the Progress of Clinical Trials for New Drug Registration in China published by CDE, NMPA.^114–117^

As illustrated own in Fig. 2, over the past five years, the number of New Drug Applications (NDAs) of innovative drug accepted by the NMPA has significantly increased.^114–117^ The annual number of accepted applications has escalated from 30 in 2019 to 133 in 2023, with a CAGR of 45.11% (Fig. 2a). The number of approved innovative drugs also significantly uptick, with the annual number of approvals rising from 20 in 2019 to 66 in 2023 (Fig. 2a). In terms of variety number of drugs, 138 innovative drug varieties were approved in total over the 5-year period, with the annual number of approvals increasing from 10 products in 2019 to 40 products in 2023, resulting in a CAGR of 41.42% (Fig. 2c).

The most notable increase in innovative drug registrations over the past five years was observed in biologics, which had a CAGR of 66% (Fig. 2b). Biologics also experienced a significant increase in approved innovative drugs, exhibiting a CAGR of 68% (Fig. 2c). In 2023, a total of 40 innovative drug varieties were approved for marketing, including 19 chemical drugs, 16 biologics, and 5 traditional Chinese medicines (Fig. 2c). In terms of marketing authorization holder (MAH) status, 90.0% were held by domestic entities. From the perspective of therapeutic areas, the most prevalent among innovative drugs approved in 2023 was anti-cancer drugs, with 14 products, double the number approved in 2022. This was followed by anti-infective drugs, with 6 products. Additionally, two TCM products for dermatology and otorhinolaryngology were approved, along with one product each in the fields of gastroenterology, respiratory medicine, and neuropsychiatry.^95^

A comprehensive inventory of Category 1 innovative drugs approved by the NMPA from 2018 to 2023 is shown in Table S1. In the first half of 2024 (January to June), according to data from the NMPA, 26 new drugs were approved, including 15 chemical drugs, 8 biologics, and 3 TCM products (Table S2). This represents an increase from the corresponding period in 2023 (24 drugs).

The rapid growth in NDA applications and approvals for innovative drugs in China underscores the significant strides made in the country’s pharmaceutical sector over the past five years. Biologics have emerged as the leading category driving this growth, with a remarkable annual growth rate. Additionally, oncology drugs dominate the therapeutic focus of approved NDAs, reflecting global trends in prioritizing high-burden and unmet medical needs. This aligns with China’s broader strategy of fostering domestic innovation to meet public health challenges, while also position China as a growing force in the global pharmaceutical landscape.

The FDA consistently led the global regulatory agencies in approving innovative drugs throughout the five-year period. As shown by Fig. 2d, starting with 48 approvals in 2019,^118^ the number steadily increased, reaching 55 approvals in 2023.^1,119,120^ This highlights the FDA’s robust capacity for drug evaluation and approval, maintaining its position as the leading regulatory body globally. Additionally, the FDA’s integration of programs like Breakthrough Therapy Designation^121–126^ and Accelerated Approval^8,127–131^ pathways continues to incentivize the rapid development of treatments targeting unmet medical needs, particularly in areas such as oncology and rare diseases.^132–138^

Despite its leadership, the FDA faces challenges in sustaining approval growth. The relatively flat trajectory in innovative drug approvals may reflect constraints in the R&D ecosystem, including the high costs of drug development, and the lengthy timelines required for clinical trials. The FDA’s emphasis on robust clinical evidence reflects its commitment to ensuring the safety and efficacy of approved therapies.^139^ While this approach minimizes risk, it may also extend the timeline for novel therapies seeking approval compared to more flexible regulatory frameworks emerging in other regions. The FDA’s focus on global therapeutic impact, however, continues to position it as the gold standard in regulatory, and its emphasis on post-marketing surveillance ensures safety while supporting innovation.

The EMA has shown a moderate performance in innovative drug approvals compared to the FDA, with approvals fluctuating between 30 in 2019 and peaking at 54 in 2021, before falling to 39 in 2023 (Fig. 2d).^2,140^ This variability reflects the challenges within Europe’s regulatory framework, where diverse national systems, extended timelines, and intricate clinical trial requirements can sometimes hinder timely access to the market. Unlike the FDA, which operates under a centralized model, the EMA’s coordination across 27 member states introduces additional layers of complexity, potentially deterring global pharmaceutical companies from prioritizing the European Union (EU) as a primary launch market for new drugs.^140,141^ The impact of Brexit on the EMA functionality has led to considerable changes in its operational structure. As a result of the United Kingdom’s departure from the European Union, the EMA has faced increased challenges in regulatory coordination, particularly in terms of the alignment of approval timelines for medicines. The relocation of the EMA’s headquarters from London to Amsterdam has further compounded these difficulties, leading to delays in decision-making processes and disruptions in the approval of new drugs. These changes have necessitated new frameworks for regulatory cooperation among EU member states, thereby adding additional complexities to the approval system.^142^ The EMA in 2024 focused on accelerating approvals, conditional marketing authorizations, and international collaboration to facilitate the timely market entry of innovative medicines while ensuring their safety and effectiveness, particularly in addressing public health needs and rare diseases.^143^

However, the EMA is generally more restrictive than the FDA in drug approvals, as seen in cases like *Aducanumab*^144–149^ and *Lecanemab*.^150–155^ While the FDA often utilizes accelerated approval pathways based on surrogate endpoints, the EMA requires more robust clinical evidence demonstrating clear patient benefits. For example, the FDA approved *Aducanumab* despite concerns over its efficacy, whereas the EMA rejected it due to insufficient proof of meaningful clinical improvement.^156^ Similarly, the EMA has taken a more cautious approach with *Lecanemab*,^157^ emphasizing a thorough risk-benefit analysis before approval. This reflects the EMA’s stricter regulatory standards and focus on long-term safety.

In terms of evidence requirements, the NMPA tends to follow a regulatory approach that shares some similarities with the FDA, especially in regard to expedited approval pathways. This can sometimes involve using surrogate endpoints or more limited clinical data, particularly in areas like oncology and rare diseases. At the same time, the NMPA is also gradually refining its regulatory processes, which may involve placing greater emphasis on clinical data and safety assessments, somewhat in line with practices seen in the EMA. However, it’s worth noting that the approach of the NMPA can vary depending on the specific therapeutic area or drug under consideration. In general, while the NMPA’s regulatory approach may reflect aspects of both the FDA and EMA, it is continuously evolving to adapt to the changing landscape of drug approval and patient safety.

Building on the observations above, the comparative trends in drug approvals across China, the FDA, and the EMA reflect broader shifts in global pharmaceutical dynamics. China’s focus on biologics and oncology reflects alignment with global trends prioritizing high-burden therapeutic areas and addressing unmet medical needs. The rapid increase in innovative drug approvals by the NMPA demonstrates the impact of regulatory changes aimed at enhancing efficiency and fostering domestic innovation. These efforts have included the integration of international standards to align with global best practices while maintaining a focus on addressing the specific healthcare needs of China’s population. This dual strategy highlights China’s commitment to advancing pharmaceutical innovation and improving public health outcomes, with a growing emphasis on strengthening its role in the global regulatory landscape.

Overall, these trends indicate an intensely competitive global landscape where regulatory frameworks are increasingly required to balance the dual imperatives of fostering innovation and ensuring patient safety. China’s rapid ascent reflects its growing ambition to play a more prominent role in the global regulatory ecosystem, while the FDA and EMA face pressures to address emerging inefficiencies to sustain their established positions as benchmarks for drug evaluation and approval. These developments underscore the importance of international harmonization and collaboration in maintaining a balanced and innovative pharmaceutical ecosystem.

Looking ahead, improving regulatory processes and enhancing efficiency will be crucial for adapting to the evolving pharmaceutical landscape. For China, expanding its focus beyond the volume of approvals to include a higher proportion of globally novel, first-in-class therapies would reinforce its position as a leader in both regulatory efficiency and pharmaceutical innovation. For the FDA and EMA, continued modernization of their regulatory frameworks will be important to maintain their competitiveness, particularly in addressing the increasing complexity of biologics, gene therapies, and precision medicine. These efforts will play a critical role in shaping the future of global drug development and ensuring equitable access to groundbreaking therapies.

As global drug development becomes increasingly complex, regulatory systems must adapt to ensure timely access to new therapies. Regulatory reliance refers to the practice where smaller or developing regulatory agencies depend on the review standards and approval decisions of larger, more experienced institutions, such as the FDA and EMA, during the drug approval process. This approach not only enhances approval efficiency and avoids redundant reviews but also ensures that drugs can rapidly reach the market, especially in complex areas such as biologics, gene therapies, and precision medicine. Many countries and regions, particularly developing nations, have started to adopt this model.^158^

In the global drug regulatory system, the FDA and EMA are key leaders. They establish rigorous standards and processes, driving the standardization of global drug approvals. The review results and approval standards set by these two agencies are often used as references by other national regulatory bodies. For instance, the UK’s Medicines and Healthcare products Regulatory Agency (MHRA), after Brexit, has further strengthened its cooperation with the FDA and EMA, thereby promoting the trend of regulatory reliance and ensuring smooth drug approval processes.^159^

In China, the NMPA has gradually absorbed the advanced experiences of the FDA and EMA in its drug approval process, particularly in modernizing the drug review and approval procedures. The NMPA has also strengthened its collaboration with international regulatory agencies to improve the efficiency of drug approvals and ensure the safety and efficacy of medications. As the NMPA’s position in the global drug regulatory system continues to grow, it may further enhance its cooperation with other regulatory bodies in the future, adopting regulatory reliance models to accelerate the drug approval process and improve patient access to treatments.

In summary, regulatory reliance, as an international collaboration model, is becoming widely adopted in the global drug regulatory field. It not only facilitates efficient approval processes but also plays a crucial role in ensuring the timely market availability of innovative therapies and equitable access for patients worldwide.

## Progress and global positioning of China’s pharmaceutical innovation

### R&D investment, new technologies, and new targets in China compared to global trends

**Fig. 3:**
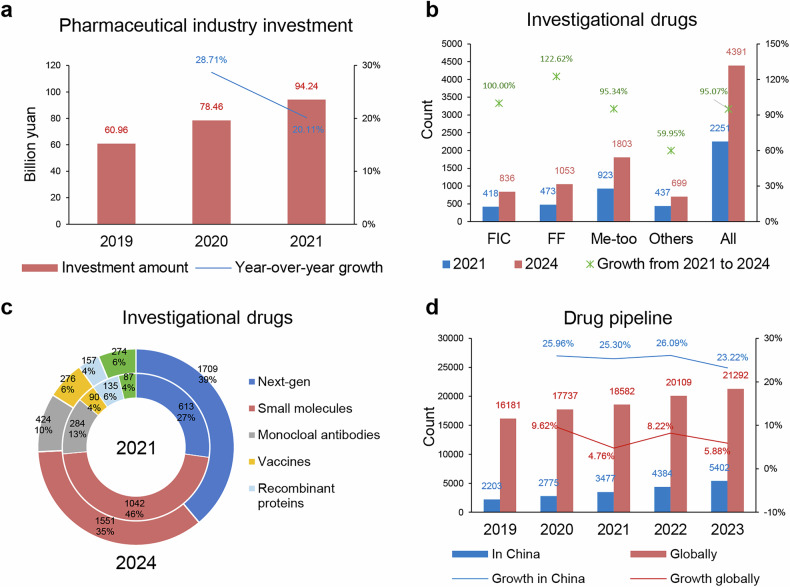
R&D investment, new technologies, and new targets in China’s innovative drugs. **a** Internal R&D expenditure in China’s pharmaceutical manufacturing industry from 2019 to 2021, along with the year-on-year growth rates.^160^ **b** Comparison of the number and growth rates of First-in-Class (FIC), Fast-Follower (FF), Me-Too and all drugs under development in China in 2021 and 2024. **c** Number and proportion of various drug types under development in China in 2021 (inner ring) and 2024 (outer ring).^44^ **d** Number of drug pipelines in China and globally from 2019 to 2023, along with annual growth rates.^177,178^

The R&D investment, pipeline layouts, and development trends of innovative drug development in China over the past five years are shown in Fig. 3. The pharmaceutical industry’s R&D investment in China has been continuously increasing.^160^ In 2019, 2020, and 2021, the internal R&D expenditures of the pharmaceutical manufacturing industry amounted to 60.96 billion yuan (approximately 9.5 billion USD), 78.46 billion yuan (approximately 12.1 billion USD), and 94.24 billion yuan (approximately 14.5 billion USD), respectively, representing a growth of 54.59% from 2019 to 2021^160^ (Fig. 3a). The R&D investment intensity of the pharmaceutical industry consistently exceeded the national average R&D investment intensity over these three years and continued to increase. Additionally, in 2022–2023, three Chinese companies, Sinopharm, Hengrui Medicine, and BeiGene, were ranked among the top 25 global pharmaceutical companies by pipeline size, demonstrating China’s emergence in global drug innovation.^160^

As of July 1, 2021, Chinese pharmaceutical companies had 2251 drugs under development across all therapeutic areas, including small molecules, monoclonal antibodies, recombinant proteins, vaccines, and next-generation drugs which encompass cell therapies, gene therapies, antibody-drug conjugates (ADCs), bispecific/multispecific antibodies, and nucleic acid-based products (Figs. 3b and c).^44^ By January 1, 2024, the number of innovative products in development by Chinese pharmaceutical companies had nearly doubled from 2251 in 2021 to 4391^161^ (Fig. 3b). Next-generation products grew rapidly, increasing from 613 to 1709 (179% growth), raising their share of the total pipeline from 27% in 2021 to 39% in 2024, surpassing the proportion of small-molecule drugs (Fig. 3c).

Based on their level of innovation, these drugs were categorized as First-in-Class (FIC), Fast-Follower (FF), or Me-Too, following internationally recognized conventions. FIC drugs refer to those that introduce a completely novel therapeutic mechanism or target, offering groundbreaking advancements in treatment and addressing unmet medical needs. FF drugs are developed after FIC drugs, leveraging similar mechanisms of action but with modifications or improvements, such as enhanced efficacy, safety, or patient convenience. Me-Too drugs, on the other hand, share the same mechanism of action as existing drugs but generally replicate these with minor modifications, focusing on market competition rather than significant innovation.^162–174^

Among the 2251 drugs, 418 were FIC, 473 were FF, and 923 were Me-Too^44^ (Fig. 3b). The growth rate of FIC drugs (increasing from 418 to 836, 100% growth) and FF drugs (increasing from 473 to 1053, 123% growth) outpaced that of Me-Too drugs (increasing from 923 to 1803, 95% growth)^161^ (Fig. 3b). Next-generation products now account for the majority of FF pipelines and FIC pipelines. Within next-generation products, cell therapies continue to dominate, while bispecific/multispecific antibodies and ADCs are the second and third largest categories.^161,175,176^ Oncology was the most active therapeutic area, accounting for 55% of all drugs, followed by infectious diseases, which made up 11%. Among FIC drugs, oncology accounted for 67% of the total.^44^

Regarding new targets and target combinations, the number of targets increased by 295 from 375 in 2021 to 670 in 2024. The integration of new technologies has also propelled a significant rise in multi-target combinations, which expanded from 207 in 2021 to 454 by 2024.^161^ An analysis of the top 10 oncology targets in 2021 and 2024 reveals that seven targets remained consistently present in both lists, with CD19 and HER2 maintaining their positions as the top two targets.^161^ A closer examination of the top ten oncology targets in these years reveals that seven of them (CD19, HER2, BCMA, PD-1, EGFR, PD-L1, CD19/CD22) have remained consistent, with CD19 and HER2 continuing to hold the top spots. Furthermore, the advent of novel therapeutic targets such as CLDN18.2, CD7, and CD276 (B7-H3) is largely propelled by the progress of cutting-edge technologies, encompassing cell therapies and ADCs. In the field of non-oncological diseases, there has been a persistent increase in candidate drugs targeting SARS-CoV-2 associated targets. Additionally, drugs targeting GLP1R, GIPR, and GCGR are progressively demonstrating promise in improving the first generation of drugs.^161^

According to Pharmaprojects data from Citeline in April 2023,^177,178^ China currently has 5402 drug pipelines (drug candidates in development), a 23.22% increase from 2022, far exceeding the global growth rate of 5.88% (Fig. 3d). Meanwhile, China is also catching up in the development of innovative drug candidates, with 1457 candidates in 2022, ranking second globally. During the same year, 73 new drugs were launched worldwide, including 74 New Active Substances (NAS). As of March 2022, China’s share of NAS is steadily increasing, with 16 NAS launched,^177,178^ accounting for 21.6% of the total new drugs.

The rapid growth in R&D investment, pipeline expansion, and technological advancements in China’s pharmaceutical sector over the past five years highlights the country’s accelerating role in global drug innovation. With R&D spending outpacing national averages and a significant increase in innovative products under development, China has emerged as a strong contender in pharmaceutical innovation. The shift towards next-generation products, such as cell therapies, ADCs, and bispecific antibodies, has reshaped the drug development landscape in China,^44,179–186^ with these cutting-edge modalities accounting for a growing share of the overall pipeline. The focus on oncology as the dominant therapeutic area, alongside the rise in FIC and fast-follower FF drugs, underscores China’s alignment with global priorities of addressing high-burden and unmet medical needs.

The significant increase in new targets and multi-target combinations further demonstrates the integration of advanced technologies into China’s drug discovery efforts. The emergence of novel targets such as CLDN18.2 and CD276, coupled with advancements in fields like antibody-based therapies and nucleic acid drugs, reflects the country’s capability to compete in the rapidly evolving pharmaceutical landscape. Furthermore, China’s contribution to the global launch of NAS is steadily increasing, accounting for 21.6% of NAS in 2022, which underscores its growing influence in introducing globally relevant therapies.

Globally, the United States continues to lead global pharmaceutical R&D, driven by its well-established infrastructure, significant private-sector investment, and focus on cutting-edge innovation. According to IQVIA’s report on global R&D trends, as of 2022, U.S.-based companies accounted for over 40% of global R&D pipelines,^187^ maintaining their dominance over the past 15 years. This leadership extends to new therapeutic modalities, with the U.S. consistently spearheading advancements in cell and gene therapies, as well as next-generation biologics, such as mRNA vaccines and ADCs. Moreover, the United States has shown resilience in sustaining investment levels despite the global economic slowdown. While total life sciences investment in the U.S. decreased by 39% from its 2021 peak, the 2022 investment level still exceeded pre-pandemic benchmarks by 25%, reflecting the sustained confidence in the country’s pharmaceutical ecosystem.^187^

In terms of innovative drug development, the United States also leads in the approval of first-in-class drugs and biologics, underpinned by regulatory pathways such as the FDA’s Breakthrough Therapy Designation and Accelerated Approval programs.^8,127–131^ These frameworks encourage the rapid development of treatments targeting unmet medical needs, particularly in areas such as oncology and rare diseases, which remain the focus of nearly half of the U.S. R&D pipeline.^8,121,123,188–195^ However, while the U.S. excels in fostering innovation and regulatory efficiency, the high costs of drug development and the increasing complexity of clinical trials remain challenges for its R&D productivity. Despite these barriers, the U.S. continues to set the benchmark for pharmaceutical innovation, reflecting its global influence in shaping the direction of drug discovery and development.

Europe’s position in global pharmaceutical R&D and innovation has faced increasing challenges, particularly in maintaining its competitive edge. Over the past decade, Europe’s share of global R&D pipelines has steadily declined, with its proportion falling from 31% in 2007 to 23% in 2022.^187^ Although the absolute number of active R&D pipelines in Europe increased from 1327 in 2007 to 1655 in 2022, this growth has been outpaced by regions such as the United States and China, where significant investments and innovation-driven policies have expanded their respective shares.^187^ This growth occurred post-Brexit, and the loss of infrastructure, such as EMA relocation from London to Amsterdam, may have had an impact on this trend.^196^ China’s share of global R&D pipelines has increased significantly over the past 15 years, rising from 2% in 2007 to 15% in 2022,^187^ demonstrating the rapid advancements made in China’s pharmaceutical R&D landscape. In addition, Europe’s average pharmaceutical investment in 2022 was approximately half of its peak in 2020. Despite some success in collaborative research initiatives and multi-country clinical trials, fragmented regulatory systems and slower processes than the U.S.^197^ have hindered Europe’s ability to compete effectively on a global scale.

In addition to the declining share of global R&D pipelines, Europe faces ongoing challenges in maintaining its competitive position in pharmaceutical innovation. While the region remains active in therapeutic areas such as oncology and rare diseases, its pipelines include a relatively smaller proportion of first-in-class therapies compared to those of the United States and China, reflecting differences in innovation focus and development strategies. Furthermore, Europe’s longer regulatory timelines and fragmented approval processes have slowed the development of next-generation therapies, including cell and gene therapies, which have experienced significant growth in the U.S. and China. At the same time, China’s progress in pharmaceutical innovation has been bolstered by increasing investments, streamlined regulatory frameworks, and an active focus on cutting-edge technologies, enabling the country to expand its global R&D presence and challenge traditional leaders in the field.

Overall, China’s pharmaceutical R&D ecosystem has experienced significant growth, marked by increases in investment, pipeline expansion, and the rapid adoption of next-generation technologies. This progress has allowed China to emerge as an important player in global drug development, particularly in innovative modalities such as cell and gene therapies, ADCs, and bispecific antibodies. While the United States continues to lead the global landscape with its well-established infrastructure, strong focus on first-in-class therapies, and dominance in advanced therapeutic areas, China’s dynamic trajectory highlights its growing capacity to reshape global innovation. In contrast, Europe, despite its historical strengths in key areas like oncology and rare diseases, faces challenges from slower regulatory timelines and fragmented systems, which have limited its competitiveness against rapidly advancing regions.^197^ These developments underline a rebalancing in global pharmaceutical R&D, as China increasingly asserts itself as a critical force shaping the future of drug innovation.

### Advances in clinical trials for new drug registration: local progress and international participation

#### Registered clinical trials for new drugs

**Fig. 4:**
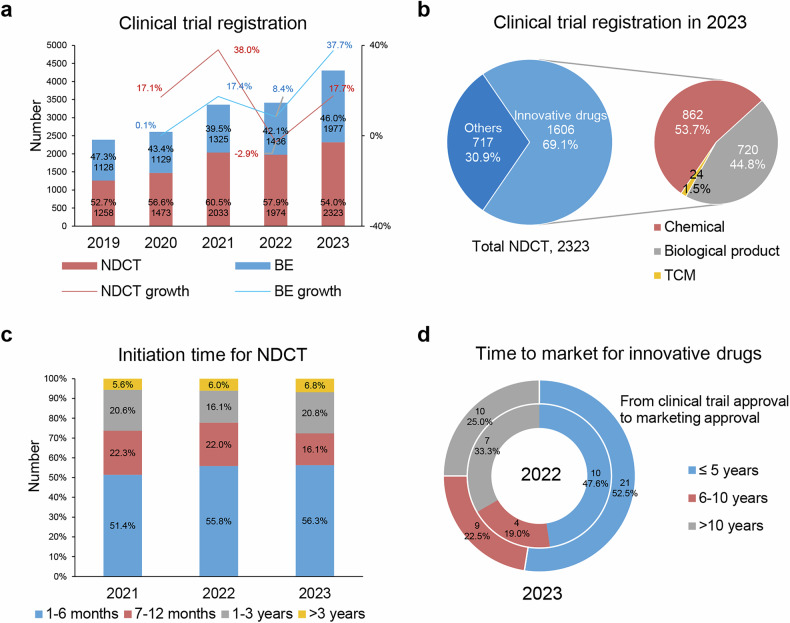
Clinical trials for new drug registration in China. **a** Number and growth rate of new drug clinical trials (NDCT) and bioequivalence (BE) trials in China from 2019 to 2023. **b** Proportion of Category 1 innovative drugs in NDCT in 2023 (left pie chart) and the proportion of chemical drugs, biological products, and TCM among Category 1 innovative drugs (right pie chart). **c** Time to initiate subject recruitment in NDCT from 2021 to 2023. **d** Time required for market approval for innovative drugs. The time to market is analyzed based on the date of the first clinical trial approval to the date of the market approval. The inner ring and outer ring of the combined ring chart represent the time to market of innovative drugs that approved for market release in 2022 and 2023, respectively. Data for this figure were derived from 2020 - 2023 Annual Report on the Progress of Clinical Trials for New Drug Registration in China published by CDE, NMPA.^114–117^

Over the past three years, the number of new drug clinical trials (NDCTs) and bioequivalence (BE) trials in China has grown year-on-year.^114–117^ In 2023, the annual number of drug clinical trial registrations in China surpassed 4000 for the first time, reaching 4300, representing a 26.1% increase compared to 2022 (Fig. 4a). In 2023, 2323 new drug clinical trials were registered (54.0%), representing an increase of approximately 17.7% compared to 2022 (1974 trials). Meanwhile, 1977 BE trials were registered (46.0%), an increase of approximately 37.7% compared to 2022 (1436 trials) (Fig. 4a).

#### Drug types, registration categories, varieties, and clinical trial phases

In 2023, among the registered new drug clinical trials, 1294 were dedicated to chemical drugs, 953 to biologics, and 76 to TCMs.^116,117^ These figures align with trends from previous years, with a slight increase in TCM trials compared to 2022. Over recent years, chemical drugs and biologics have dominated new drug clinical trials, with chemical drugs comprising over half of the total, and biologics approximately 40%. The proportion of TCM trials in 2023 was 3.3%, mirroring the percentage from 2022 (3.1%).

In terms of registration categories, a total of 1606 trials were registered for Category 1 innovative drugs in 2023, accounting for 69.1% of the overall trials (1606/2323). Chemical drugs dominated with 53.7% (862/1,606) of the registered innovative drug trials (Fig. 4b). Furthermore, there was a marked increase in the registration of clinical trials for cell and gene therapy (CGT) products, which rose to 81 in 2023, a nearly twofold increase from the 46 trials registered in 2022. These trials were predominantly concentrated on domestic Phase I anti-cancer drugs. The number of clinical trials for medical imaging drugs reached 14, the highest in recent years.

Regarding varieties and indications, clinical trials for Category 1 innovative drugs were predominantly aimed at oncology, accounting for 40.6% of the total (652/1606). As for innovative TCMs trials, they were primarily focused on five indication areas: respiratory, dermatology and otorhinolaryngology, cardiovascular, gastrointestinal, and neuropsychiatric disorders, accounting for approximately 80.3% of all TCM clinical trials. Encouraged by supportive policies for innovation, research into drugs for children, rare diseases, and niche areas such as medical imaging and radiopharmaceuticals has become more active. In terms of clinical trial phases, in 2023, there were 978 Phase I trials, 440 Phase II trials, 489 Phase III trials, and 62 Phase IV trials.^116,117^ The proportion of trials in each phase remained consistent with 2022.

Over the past three years, China’s clinical trial landscape has experienced significant growth, with a notable increase in both NDCTs and BE studies. In 2023, the total number of registered drug clinical trials in China exceeded 4000 for the first time, marking a 26.1% rise compared to 2022.^198^ This upward trend reflects China’s commitment to advancing pharmaceutical research and development. This expansion encompasses a diverse range of drug types, including chemical drugs, biologics, and TCMs. Innovative drug trials, particularly those in Category 1, have seen substantial growth, accounting for a significant portion of the overall trials. Additionally, there has been a marked increase in clinical trials for CGT products, indicating China’s dedication to pioneering advanced therapeutic modalities.

Globally, the clinical trial landscape has been evolving, with regions such as North America and China experiencing growth in trial activity. China’s share of global clinical trial activity reached 27.7% by 2022, reflecting its increasing prominence in the field.^199–201^ This shift underscores the dynamic nature of global clinical research and the varying regional focuses on different therapeutic areas and phases of clinical development.

When comparing China’s clinical trial growth to global trends, several key distinctions emerge. China has experienced a significant increase in early-phase trials, particularly Phase I studies, underscoring its commitment to innovative drug development. This expansion is largely driven by domestic sponsors, who accounted for 91.7% of new registrations in 2023.^198^ China’s increasing participation in global multicenter studies reflects its deeper integration into the international clinical research community. This involvement contributes to the diversification and globalization of clinical data, enhancing the robustness and applicability of research outcomes worldwide.

In summary, China’s clinical trial landscape has experienced significant growth, with a notable increase in both new drug clinical trials and bioequivalence studies. This growth positions China as a significant player in the global clinical trial landscape, contributing to the diversification and globalization of clinical data. However, challenges remain, including the acceptability of clinical trials conducted solely in China by regulatory authorities in the USA and Europe. For instance, Sintilimab, a PD-1 inhibitor, was approved in China but faced rejection by the FDA due to the reliance on data from the ORIENT-11 trial, which was conducted exclusively in China.^202^ This case highlights the need for more diverse, multiregional clinical trials to meet international regulatory standards. Addressing these challenges will be crucial for China’s continued advancement in global clinical research.

#### Efficiency of clinical trial registration, review, and implementation

Clinical trial initiation and execution in China have demonstrated a marked improvement in efficiency. During the years 2021, 2022, and 2023, over half of the new drug clinical trials initiated subject recruitment within a six-month timeframe. In 2023, the proportion of trials that commenced subject recruitment within six months slightly increased to 56.3%, exhibiting a slightly upward trend from the 51.4% in 2021 (Fig. 4c). An analysis of the geographical distribution of clinical trial sites revealed that trials conducted in areas with a higher density of leading sites exhibited a longer initiation period.

Upon analyzing the initiation of subject recruitment in the same year as the trial approval, 2023 exhibited a further reduction in initiation time compared to 2022, with an average time of 3.0 months (down from 3.3 months in 2022). The proportion of trials that initiated recruitment within six months then increased to 93.4% in 2023 (from 91.5% in 2022).

Analyzing upon examination of the interval from the initial approval of domestic clinical trials to the ultimate grant of marketing authorization for innovative drugs, the average time to market for innovative drugs approved in 2022 was 7.6 years, and in 2023 was 7.2 years. Among the innovative drugs approved for marketing in 2022, 10 products (47.6%) were brought to market within five years. In 2023, 21 innovative drugs (52.5%) were marketed within five years (Fig. 4d). These findings suggest a trend towards accelerated drug development and regulatory processes, facilitating faster patient access to novel therapeutics.

China has made significant strides in enhancing the efficiency of its clinical trial processes. Recent changes have streamlined the approval timelines for clinical trial applications (CTAs). Previously, obtaining CTA approval could take two to three years; however, with the implementation of a 60-working-day “silent approval” mechanism,^30,203–205^ applications are now approved by default if no response is received within this period. This acceleration has led to a notable increase in the number of innovative drugs entering the market.

The U.S. FDA has established review timelines for NDAs. Standard reviews are typically completed within 10 months, while priority reviews are expedited to a six-month timeframe.^206,207^ The FDA offers expedited programs, such as Fast Track, Breakthrough Therapy, Accelerated Approval, and Priority Review, to facilitate the development and review of drugs addressing unmet medical needs.^8,208^ These programs can significantly reduce the time to market for critical therapies.

The EMA follows a centralized procedure for marketing authorization applications, with a standard review timeline of up to 210 active days, excluding clock stops when applicants prepare responses to questions.^209^ The EU’s Clinical Trials Regulation, which came into application on 31 January 2022, aims to harmonize the assessment and supervision processes for clinical trials throughout the EU via the Clinical Trials Information System (CTIS).^105,210^ This regulation seeks to streamline procedures and improve the environment for conducting clinical research across member states.

China’s recent changes have led to notable improvements in the efficiency of clinical trial processes, with reduced approval times and quicker trial initiations. The FDA maintains structured and predictable review timelines, with mechanisms to expedite reviews for priority medicines. The EMA’s centralized procedure offers a harmonized approach across EU member states, though its standard review timeline is longer compared to the FDA’s priority review process. While the FDA and EMA have established expedited pathways for critical therapies, China’s implementation of the “silent approval” mechanism represents a distinctive approach to accelerating clinical trial approvals.

In summary, each region has developed regulatory strategies to balance the need for timely access to new therapies with the imperative of ensuring safety and efficacy. China’s recent changes reflect a commitment to enhancing efficiency in clinical trial processes, positioning it as an increasingly significant player in the global pharmaceutical landscape.

## Notable innovative drugs in China and international market

As delineated in Table 2, the past 5 years have seen the inaugural approvals and continuous updates, including new indications, improved formulations, and line extensions, of therapeutic drugs in the fields of major public health diseases, malignant tumors, and other life-threatening or quality-of-life-impacting conditions. This has continuously driven the launch of new and high-quality drugs in the fields of rare diseases and pediatric medicine, meeting the clinical medication needs of patients. Notably, the number of vaccine products has significantly increased, ensuring diversity and coverage through a variety of technological approaches, including inactivated vaccines, adenovirus vector vaccines, and recombinant protein vaccines, providing effective prevention against diseases with significant public health impacts. Important breakthroughs have also been made in other vaccine areas, such as rotavirus and influenza vaccines, enriching the domestic vaccine portfolio and technological pathways, thereby enhancing public health prevention and control capabilities.

**Table 2:** Notable innovative drugs approved in China in the past five years

Product name	Time of approval	Manufacturer	Indication	Remark
Chemical and Biotherapeutic Drugs				
Benvitimod Cream	05/31/2019	Guangdong Zhonghao	Mild to moderate stable plaque psoriasis in adults	Approved through the priority review and approval process.
Ormutivimab Injection	01/25/2022	North China Pharmaceutical	Passive immunization for adults exposed to rabies virus	Approved through the priority review and approval process; First recombinant human anti-rabies monoclonal antibody in China.
Tafolecimab Injection	08/16/2023	Innovent Biologics (Suzhou)	Primary hypercholesterolemia and mixed dyslipidemia in adults	First domestically developed PCSK9 monoclonal antibody inhibitor in China.
Surufatinib Capsules (SULANDA)	12/30/2020	Hutchison Medipharma (Shanghai)	Neuroendocrine tumors	Approved through the priority review and approval process.
Narlumosbart Injection	09/06/2023	Shanghai JMT	Giant cell tumor of bone in adults	Conditionally approved; First domestically developed RANKL-targeted drug.
Sunvozertinib Tablets	08/23/2023	Dizal (Jiangsu)	Locally advanced or metastatic non-small cell lung cancer in adults	Conditionally approved; First domestic tyrosine kinase inhibitor targeting EGFR exon 20 mutations.
Simnotrelvir Tablets/Ritonavir Tablets (co-packaged)	01/29/2023	Simcere (Hainan)	Mild to moderate COVID-19 in adults	Emergency review and conditional approval; Meets dynamic needs of China’s COVID-19 prevention efforts.
Deuremidevir Hydrobromide Tablets	01/29/2023	Shanghai Wangshi	Mild to moderate COVID-19 in adults	Emergency review and conditional approval; Supports China’s COVID-19 prevention efforts.
Leritrelvir Tablets	03/23/2023	Guangdong Zhongsheng Ruichuang	Mild to moderate COVID-19 in adults	Conditionally approved; First 3CL protease inhibitor approved in China.
Coblopasvir Hydrochloride Capsules	02/12/2020	Beijing Kain-Gelin	Chronic hepatitis C virus (HCV) infection in adults	Approved through the priority review and approval process; First domestically developed broad-spectrum anti-HCV drug.
Ainuovirine	06/23/2021	Jiangsu Hansoh	Chronic hepatitis B in adults	Approved through the priority review and approval process; First domestically developed small-molecule anti-hepatitis B drug.
Ainomeptide Tablets	01/04/2023	Jiangsu Aide	HIV-1 infection in treatment-naïve adults	First domestically developed novel anti-HIV combination therapy.
Spesolimab-sbzo Injection	12/14/2022	Boehringer Ingelheim International GmbH	Generalized pustular psoriasis in adults	Approved through the priority review process; Global simultaneous development and launch.
Ritlecitinib Tosylate Capsules	10/19/2023	Pfizer	Severe alopecia areata in adolescents and adults (12 years and older)	Approved through the priority review process; First drug in China for adolescent alopecia areata; global simultaneous development and launch.
Risdiplam Oral Solution	06/17/2021	Roche	Spinal muscular atrophy (SMA) in patients 2 months and older	Approved through the priority review process; Global simultaneous development and launch; First oral treatment for SMA.
Siponimod Tablets	05/11/2020	Novartis Pharma AG	Relapsing multiple sclerosis in adults	Approved through the priority review process; Global simultaneous development and launch.
Vericiguat Tablets	05/19/2022	Bayer	Chronic heart failure in adults	Approved through the priority review process; Global simultaneous development and launch.
Pralsetinib Capsules	03/24/2021	Blueprint Medicines Corporation	Locally advanced or metastatic non-small cell lung cancer in adults (NSCLC)	Conditionally approved through the priority review process; RET (Rearranged during Transfection) inhibitor.
Glofitamab Injection	11/08/2023	Roche Pharma (Schweiz) AG	Relapsed or refractory diffuse large B-cell lymphoma in adults	Conditionally approved through the priority review process; First bispecific CD3/CD20 antibody approved in China; globally co-developed and launched.
Vaccines				
Inactivated COVID-19 Vaccine (Vero Cell)	12/30/2020	Sinopharm CNBG Beijing	Prevention of COVID-19	Emergency review and conditional approval; First domestically approved inactivated COVID-19 vaccine.
Inactivated COVID-19 Vaccine (Vero Cell)	02/05/2021	Sinovac Biotech (Beijing)	Prevention of COVID-19	Emergency review and conditional approval; Second domestically approved COVID-19 vaccine.
Recombinant COVID-19 Vaccine (Adenovirus Type 5 Vector)	02/25/2021	CanSino Biologics	Prevention of COVID-19	Emergency review and conditional approval; First domestically approved adenovirus vector COVID-19 vaccine.
Inactivated COVID-19 Vaccine (Vero Cell)	02/25/2021	Sinopharm CNBG Wuhan	Prevention of COVID-19	Emergency review and conditional approval.
Recombinant COVID-19 Protein Vaccine (CHO Cell)	03/01/2022	Anhui Zhifei Longcom	Prevention of COVID-19	Emergency review and conditional approval; First globally approved recombinant protein COVID-19 vaccine; First overseas clinical trial inspected by Chinese regulators.
Oral Trivalent Live Attenuated Rotavirus Vaccine (Vero Cell)	04/17/2023	Sinopharm CNBG Lanzhou	Prevention of infant diarrhea caused by rotavirus (types G1, G2, G3, G4, G9)	First domestically developed trivalent rotavirus vaccine.
Quadrivalent Influenza Virus Subunit Vaccine	05/12/2023	Jiangsu Zhonghui Yuantong	Influenza virus infection in individuals aged 3 years and older	First domestically developed quadrivalent influenza virus subunit vaccine.
Cell and Gene Therapy				
Relmacabtagene Autoleucel Injection	09/01/2021	Shanghai, JW Therapeutics	Relapsed or refractory large B-cell lymphoma in adults	First domestically developed and approved Category 1 cell therapy product in China.
Inaticabtagene Autoleucel Injection	11/07/2023	Tianjin Juventas Cell Therapy	Relapsed or refractory B-cell acute lymphoblastic leukemia in adults	Conditionally approved through the priority review process.
Equecabtagene Autoleucel Injection	06/30/2023	Nanjing IASO Bio	Relapsed or refractory multiple myeloma in adults	Conditionally approved through the priority review process.
TCM Innovative Drugs				
*Ramulus Mori* (Sangzhi) alkaloids tablets	03/27/2020	Beijing Wehand-Bio	Type 2 diabetes	First anti-diabetic TCM drug approved in the last decade.
Icariin Soft Capsules	01/10/2022	Beijing Shenogen	Hepatocellular carcinoma	Conditionally approved through the priority review process; Class 1.2 TCM innovative drug.
Yiqi Tongqiao Pills	09/14/2021	Tianjin Dongfang Huakang	Seasonal allergic rhinitis (lung and spleen qi deficiency syndrome)	TCM compound preparation based on clinical experience.

CGT products have emerged as innovative therapeutic options for an array of refractory and rare diseases. Several domestically developed CGT products have been approved for market, marking significant breakthroughs in this frontier field in China. The national regulatory framework, through the priority review and approval process, has facilitated the earlier market entry and clinical utilization of CAR-T products for the treatment of relapsed/refractory adult acute lymphoblastic leukemia and relapsed/refractory multiple myeloma to enter the market and clinical application earlier, allowing patients to benefit sooner.

China’s drug approvals are increasingly aligned with those in the US and Europe due to the adoption of international standards, accelerated approval pathways for globally approved drugs, and participation in global clinical trials. Simultaneous or near-simultaneous approvals are becoming more common, reflecting China’s integration into the global regulatory landscape. Additionally, local innovation is now influencing global markets, while regulatory reforms have reduced the need for duplicative trials, enabling greater reliance on global data.^211,212^

In this section, we will focus on China’s recent advancements in pharmaceutical innovation, emphasizing key breakthroughs across various drug categories, including chemical drugs, therapeutic biologics, cell and gene therapies, and vaccines. These categories are further subdivided into therapeutic areas, such as oncology, cardiovascular diseases, public health diseases, and rare diseases. We will explore China’s progress in each area, providing a comprehensive overview of its achievements in drug development. In parallel, we will also discuss notable innovations from global markets, primarily focusing on advancements in the U.S. and Europe. This allows us to highlight not only China’s pharmaceutical progress but also the global context in which these advancements are unfolding. The section will culminate in a comparative analysis between China and the leading global players, examining the similarities and differences in drug development approaches, regulatory processes, and innovations.

For each of the categories mentioned, we will present a selection of significant drugs that showcase both China’s innovations and global advancements. These drugs were chosen based on their innovative mechanisms, their ability to address critical medical needs, and their regulatory achievements, such as being first-in-class or receiving expedited approvals. By highlighting these examples, we aim to emphasize the diversity and global nature of pharmaceutical progress, illustrating how innovations in China are aligned with and contribute to global healthcare. The selected drugs reflect key challenges and accomplishments in drug development, offering insight into the evolving landscape of pharmaceutical R&D across various regions. The diseases or conditions involved below are characterized by the emergence of novel therapeutic options, the potential for significant clinical benefit, or a large unmet medical need. This approach allows for a comprehensive comparison of the most impactful developments in the pharmaceutical sector, both in China and globally.

### China’s highlights in innovative drugs

#### Chemical drugs

##### Psoriasis Treatment

First, in the field of psoriasis treatment, China has pioneered the approval of *Benvitimod Cream* (Table 2), the world’s first topical treatment for plaque psoriasis in nearly 20 years on a global scale. This drug is a Category 1 innovative drug of Chinese origin, characterized by a novel structure and mechanism of action, with independent intellectual property and multiple patents.^213–218^ Initially approved in China, its approval journey highlights the effectiveness of China’s regulatory pathways. The drug underwent a priority review by the NMPA, which expedited its evaluation process due to its innovative nature and the unmet medical need it addressed. After receiving approval in China, it was subsequently approved by the FDA in the United States three years later, marking an important milestone in international recognition. This regulatory process not only underscores the NMPA’s alignment with global standards but also demonstrates China’s ability to facilitate the approval of novel drugs that meet rigorous international criteria. The successful regulatory approval in both China and the U.S. further emphasizes *Benvitimod*’s role as a safe, effective treatment for psoriasis, fulfilling the critical gap in treatment options and showcasing China’s growing influence in global pharmaceutical innovation.

##### Cardiovascular Disease Management

*Tafolecimab Injection*, approved in 2023, was selected as a key example due to its innovative nature as the first domestically developed PCSK9 monoclonal antibody inhibitor in China. This drug addresses significant unmet medical needs in the management of dyslipidemia and its related complications, particularly in patients who struggle to achieve desired lipid levels with conventional treatments.^219–224^ Tafolecimab’s innovative mechanism of action, targeting PCSK9, provides a more effective approach to controlling LDL-C levels, a major risk factor for atherosclerotic cardiovascular disease (ASCVD). The drug’s approval is a reflection of China’s increasing ability to develop first-in-class therapies, and it has been selected for its clinical relevance, regulatory milestone, and potential impact on public health. Moreover, Tafolecimab’s dosing regimen, requiring only a subcutaneous injection every 6 weeks, significantly improves patient compliance, making it a practical and accessible treatment option for a broader patient population. This combination of clinical efficacy and regulatory success exemplifies China’s growing leadership in innovative drug development.

##### Oncology

In the realm of oncology, *Surufatinib Capsules* represent a novel, oral multi-targeted, anti-angiogenic small-molecule tyrosine kinase inhibitor (TKI), and the first innovative drug approved in China for the treatment of unresectable locally advanced or metastatic, progressive, well-differentiated (G1, G2), non-pancreatic neuroendocrine tumors. This product has demonstrated significant efficacy, markedly reducing disease progression and mortality risk in these patients, filling a treatment gap in this disease area upon its approval.^225–235^ *Surufatinib* is highlighted for its novel mechanism and ability to address a significant unmet need in a challenging disease area.

*Sunvozertinib Tablets* were conditionally approved in 2023 for the treatment of adult patients with locally advanced or metastatic non-small cell lung cancer (NSCLC) harboring *epidermal growth factor receptor (EGFR) exon 20* insertions, who have experienced disease progression during or after platinum-based chemotherapy, or who are intolerant to platinum-based chemotherapy.^236–246^ Approximately 2% of NSCLC patients carry *EGFR exon 20 insertion* mutations. The predominant clinical treatment for these patients is platinum-based chemotherapy, however, the pursuit of enhanced therapeutic outcomes remains a critical area of focus.^247^ This innovation not only expands the therapeutic landscape for NSCLC patients with this specific genetic aberration but also underscores the ongoing commitment to improving the efficacy and accessibility of targeted therapies for this patient population (Table 2). *Sunvozertinib* is highlighted due to its targeted precision for a specific mutation and its potential to improve outcomes for patients with limited treatment options.

*Pralsetinib Capsules* were approved in 2021 for the treatment of adult patients with *RET fusion-positive* locally advanced or metastatic NSCLC who have previously undergone platinum-based chemotherapy. This product is a receptor tyrosine kinase *RET* (Rearranged during Transfection) inhibitor that selectively inhibits RET kinase activity and dose-dependently inhibits RET and its downstream molecular phosphorylation, effectively suppressing the proliferation of cells expressing *RET* (both wild-type and various mutant types).^248–254^ The approval of this drug provides a new treatment option for patients. *Pralsetinib* is highlighted for its targeted action on RET fusions, a key genetic driver of NSCLC, offering a novel solution to patients with otherwise limited treatment options.

##### COVID-19 Therapies

In response to the global public health emergency caused by COVID-19, three domestically developed Class 1 innovative drugs have been approved: *Simnotrelvir Tablets/Ritonavir Tablets (co-packaged)*,^255–261^ *Deuremidevir Hydrobromide Tablets*,^255,262–264^ and *Leritrelvir Tablets*,^265–268^ all intended for the treatment of mild to moderate COVID-19 in adult patients. These drugs are highlighted for their innovative mechanisms of action, addressing the urgent need for effective treatments during the COVID-19 pandemic. They offer supportive data showing sensitivity to current circulating variants, meeting the dynamic needs of China’s pandemic prevention efforts. Notably, *Leritrelvir Tablets* is the first domestically approved single-drug 3CL protease inhibitor in China. Its rapid approval has significantly alleviated the limitations of 3CL protease inhibitors that require combination therapy, providing more treatment options for elderly patients on concomitant medications and those with high-risk factors.

##### Public Health Diseases

To address unmet clinical needs in major public health diseases in China, the NMPA has also approved four drugs for the treatment of chronic hepatitis C virus (HCV) infection, three drugs for HIV infection, and one drug for chronic hepatitis B, all of which are domestically developed Class 1 innovative drugs. Among them, *Coblopasvir Hydrochloride Capsules*^269–272^ is the first domestically developed broad-spectrum anti-HCV drug, *Tenofovir Amibufenamide Tablets*^273–280^ is the first domestically developed small-molecule anti-HBV drug, and *Ainuovirine*^281–288^ is the first domestically developed novel anti-HIV combination therapy, providing patients with more treatment options. These drugs are highlighted here for their groundbreaking roles in addressing critical public health needs. Their approval processes were expedited through the NMPA’s priority review and approval pathways, allowing for faster access to these much-needed therapies and enhancing their availability to patients in China.

#### Therapeutic biologics

##### Rabies Prevention

In the prevention and control of rabies, *Ormutivimab Injection* was approved in 2022 for passive immunization in patients exposed to the rabies virus. Rabies, caused by the rabies virus, has an almost 100% fatality rate and is a major public health threat in China (Table 2). According to the WHO consensus, passive immunization for rabies is primarily aimed at patients with suspected rabies virus exposure (Category III). Existing passive immunization agents, such as immunoglobulins or sera, have limited supply, high costs, potential risks of serum sickness, and blood-borne disease transmission.^289–293^ *Ormutivimab* is highlighted for being the first recombinant human anti-rabies monoclonal antibody developed domestically in China, offering a critical alternative to traditional passive immunization agents that are limited by supply, cost, and safety risks. The approval of *Ormutivimab* followed a rigorous evaluation process, with the NMPA granting it Category 1 innovative drug status due to its novel therapeutic approach. The expedited approval was facilitated by the drug’s potential to address a public health crisis, ensuring rapid availability for patients exposed to the rabies virus.

##### Oncology

*Narlumosbart Injection* received conditional approval in 2023 for adult patients with giant cell tumor of bone (GCTB) that is unresectable or where resection could result in severe functional impairment.^294,295^ GCTB is an aggressive tumor characterized by osteolytic destruction, heavily enriched with receptor activator of nuclear factor kappa-B ligand (RANKL), leading to bone destruction, deformity, and disability.^296^ Surgery is the main treatment for GCTB, but some cases are considered “difficult to operate on (resection may cause severe functional impairment or complications)” or “unresectable,” leading to significant unmet clinical needs. This product is the first domestically developed RANKL-targeted drug, which blocks the interaction between RANKL and its receptor RANK, inhibiting osteoclast development and suppressing bone resorption. The introduction of *Narlumosbart Injection* has expanded the therapeutic armamentarium for GCTB, offering an alternative treatment option for patients who may not be candidates for surgery.

*Glofitamab Injection* has been granted conditional approval in 2023 for the treatment of adult patients diagnosed with relapsed or refractory (R/R) diffuse large B-cell lymphoma (DLBCL) who have received at least systemic therapy regimens. DLBCL is the most common subtype of B-cell-derived non-Hodgkin lymphoma, with an estimated 40% of patients experiencing R/R, and there are currently no effective treatment options available. The clinical demand for alternative therapies is significant. This product, submitted for approval in parallel in China, the US, and Europe, is the first CD3 and CD20 bispecific antibody approved in China. It functions by binding to CD20 expressed on the surface of target B cells and the CD3ε chain (CD3ε) on effector T cells, thereby inducing T cell activation, proliferation, cytokine release, and tumor cell lysis, providing a new treatment option for R/R DLBCL patients.^297–306^

##### Rare Diseases

*Spesolimab-sbzo Injection* was approved in 2022 for the treatment of Generalized Pustular Psoriasis (GPP) in adults. GPP is a life-threatening disease with no effective treatment and has been included in China’s list of rare diseases. The rapid and sustained clearance of pustules and improvement in skin symptoms by this product met urgent patient needs.^307–310^

*Ritlecitinib Tosylate Capsules* was approved in 2023 for the treatment of severe alopecia areata in adolescents aged 12 and older, as well as in adults. Alopecia areata is a specific autoimmune disease with a rising prevalence, especially among younger populations. The incidence rate among children and adolescents is as high as 12.8%. Although the condition is not life-threatening, long-term hair loss can severely affect a patient’s psychological health and quality of life, particularly in adolescents. This drug has shown significant efficacy in improving the severity of hair loss and overall patient response.^311–315^ It is the first medication approved in China for the treatment of alopecia areata in adolescents, addressing an urgent need in this patient group. The drug was developed and launched simultaneously on a global scale.

*Risdiplam Oral Solution* was approved in 2021 for the treatment of spinal muscular atrophy (SMA) in patients aged 2 months and older. SMA is listed in the “First Batch of Rare Disease Lists” published by China’s National Health Commission. The disease typically manifests before 6 months of age, with a life expectancy often not exceeding 2 years. This product is the first oral medication for SMA, enabling patients to achieve unsupported sitting, reach motor milestones, significantly improve survival, and avoid permanent mechanical ventilation.^316–321^ Developed and launched globally simultaneously, this drug meets the urgent needs of pediatric SMA patients in China.

*Siponimod Tablets* were approved in 2020 for the treatment of adult relapsing forms of multiple sclerosis, including clinically isolated syndrome, relapsing-remitting disease, and active secondary progressive disease. Multiple sclerosis has been included in the “First Batch of Rare Disease Lists” published by China’s National Health Commission, with a high rate of disability and mortality. This product offers a new treatment option for patients with relapsing forms of multiple sclerosis in China and was developed and launched globally simultaneously.^322–331^

##### Heart Failure

*Vericiguat Tablets* were approved in 2022 for the treatment of adult patients with symptomatic chronic heart failure with reduced ejection fraction (ejection fraction <45%) who are stabilized after recent decompensation requiring intravenous therapy, to reduce the risk of heart failure hospitalization or the need for emergency intravenous diuretic treatment. Symptomatic chronic heart failure with reduced ejection fraction severely affects the daily functioning and quality of life of patients, with a heavy economic burden and high mortality rate. This product was developed and launched globally simultaneously, providing a new treatment option for heart failure patients in China.^248,332–339^

#### Cell and gene therapy products

Cell and gene therapies (CGT) have emerged as a major focus in global biopharmaceutical innovation, offering transformative solutions for previously untreatable and rare diseases.^340^ Internationally, regions like the United States and Europe have led advancements in CGT, with numerous products receiving accelerated approvals, reflecting the increasing prioritization of these therapies. Similarly, China has made significant strides in this domain, leveraging policy-driven innovation and expedited regulatory pathways to foster the development of groundbreaking CGT products. This progress underscores China’s growing integration into the global CGT landscape, demonstrating its capacity to contribute to and compete within the international biopharmaceutical ecosystem.

The development of products related to CGT is currently a key area of focus in international medical frontiers, offering new options and hope for the treatment of various diseases, particularly for difficult-to-treat and rare conditions. *Relmacabtagene Autoleucel Injection* (Table 2) is the first domestically developed Category 1 cell therapy product approved for market in China. It is used to treat adult patients with relapsed or refractory large B-cell lymphoma (including diffuse large B-cell lymphoma not otherwise specified, primary mediastinal large B-cell lymphoma, high-grade B-cell lymphoma, and diffuse large B-cell lymphoma from follicular lymphoma) who have previously received at least two lines of systemic therapy.^341–345^ *Relmacabtagene Autoleucel Injection* is an autologous immune cell injection, prepared from autologous CD19-targeted CAR-T cells genetically modified by a lentiviral vector carrying the CD19 CAR gene. The approval of this product fills a gap in domestically developed Category 1 cell therapy products, providing a new treatment option for adult patients with relapsed or refractory large B-cell lymphoma after second-line or higher systemic treatments.

In 2023, China also approved two additional domestically developed CAR-T products, namely *Inaticabtagene Autoleucel Injection* and *Equecabtagene Autoleucel Injection*. *Inaticabtagene Autoleucel Injection* is used to treat relapsed/refractory B-cell acute lymphoblastic leukemia in adults while *Equecabtagene Autoleucel Injection* is indicated for the treatment of adult patients with relapsed/refractory multiple myeloma.^346–350^ The market launch of these two CAR-T cell therapy products not only enhances the cure rates for these advanced hematological malignancies and significantly improves patient outcomes but, more importantly, represents a major breakthrough in the approval of domestically developed CAR-T products in China. This achievement holds milestone significance in the research and industrialization of this cutting-edge field of biopharmaceuticals.

The national regulatory approval system has played a crucial role in promoting the development and market entry of CGT products. Both *Inaticabtagene Autoleucel Injection* and *Equecabtagene Autoleucel Injection* were conditionally approved through the priority review and approval process, which accelerated the evaluation and approval of these innovative drugs, shortening their time to market. This allowed these CAR-T products to enter the market earlier and be applied clinically, offering new treatment options for patients. Additionally, the NMPA has provided policy support and guidance, helping domestic companies overcome various challenges in research, development, and approval processes, promoting the rapid advancement of domestically developed products. These measures have collectively driven innovation and progress in the field of CGT, accelerating the clinical application of new therapies and benefiting more patients.

#### Vaccine

China’s COVID-19 vaccine products have demonstrated significant advancements, with vaccines developed along five different technical routes having received approval for market release.^351–354^ As shown in Table 2, the NMPA granted conditional approval for the *Sinopharm CNBG Beijing inactivated COVID-19 vaccine (Vero cells)* on December 30, 2020, through an emergency review and approval process. This vaccine was the first Chinese-made inactivated COVID-19 vaccine to receive approval.^355–357^ The second vaccine to be approved for market release was the CoronaVac inactivated vaccine (Vero cells) from Sinovac.^358–361^ This vaccine is currently the most exported COVID-19 vaccine from China, having been granted emergency use or conditional market approval in over 50 countries, and is listed on the WHO Emergency Use Listing (EUL). It is the only domestically produced COVID-19 vaccine supplied to the Hong Kong region and has been recognized by Hong Kong authorities in comparative studies with the Pfizer mRNA COVID-19 vaccine. The recombinant adenovirus type 5 vector COVID-19 vaccine (CanSino) was the first domestically produced adenovirus vector COVID-19 vaccine to receive approval.^362–365^

Since May 2021, the above three vaccines have been successively certified by the WHO and included in the EUL. The Sinopharm Wuhan COVID-19 inactivated vaccine (Vero cells) also received conditional approval through an emergency review and approval process. The Anhui Zhifei Longcom recombinant protein COVID-19 vaccine (CHO cells) was the first recombinant protein COVID-19 vaccine to be approved globally and the first in China to be approved for use in children aged 3–17.^366–368^ Additionally, this vaccine was the first and so far the only one to undergo overseas clinical trial site inspection by Chinese regulatory authorities.

Moreover, other vaccine products have also received approval. As shown On April 17, 2023, the oral trivalent rotavirus attenuated live vaccine (Vero cells) developed by Sinopharm Lanzhou was approved. This marks China’s first domestically developed polyvalent rotavirus vaccine, verified for safety and efficacy through protective efficacy trials, and currently the only one available on the market in China.^369,370^ The rotavirus vaccine is a WHO priority prequalification (PQ) vaccine, and globally, there are only two other polyvalent rotavirus vaccines in production, by Merck and in India. Additionally, in May 2023, the quadrivalent influenza virus subunit vaccine from Jiangsu Zhonghui Yuantong was approved, becoming the first domestically produced quadrivalent influenza virus subunit vaccine in China.^371^ It is intended for the prevention of influenza caused by the virus types included in the vaccine for children aged three years and above and adults, adding diversity to the technical routes of influenza vaccines available in China and expanding the range of vaccination options.

In summary, there has been substantial progress in China regarding the research and approval of vaccine products, attributed to its continuously improving innovative drug research capabilities and the support and facilitation provided by the national regulatory and approval systems. Through a diverse range of COVID-19 vaccine technologies, including inactivated vaccines, adenovirus vector vaccines, and recombinant protein vaccines, China has ensured the diversity and coverage of its vaccine products.^372^ Several vaccines have gained global recognition, with some being the first of their kind to receive approval worldwide, showcasing China’s innovation capacity and international influence in the biopharmaceutical sector.^372,373^

The NMPA’s special approval procedures have accelerated the emergency review and approval of COVID-19 vaccines, ensuring that they are prioritized and made available quickly in urgent situations. China’s regulatory agencies have also conducted overseas clinical trial inspections, enhancing the international credibility of these vaccines. Beyond COVID-19 vaccines, China has developed a diverse range of vaccine products, with increasing focus on innovation and enhancing domestic public health prevention. Advancements in vaccine research and approval processes in China have been observed in the biopharmaceutical field. These developments have been aligned with both domestic public health needs and global public health initiatives.

### Global highlights in innovative drugs

#### Chemical drugs

##### Oncology

In 2021, Sotorasib became the first KRAS G12C inhibitor to gain approval, revolutionizing the treatment of NSCLC.^374–393^ The KRAS gene had long been considered an “undruggable” target, and the approval of Sotorasib marked a significant milestone in precision medicine, offering hope in an area previously deemed unattainable.

Approved by the FDA in 2020 under the accelerated approval pathway, Lurbinectedin provides a novel treatment option for small cell lung cancer (SCLC).^394–413^ Its unique mechanism of action targets transcriptional pathways, addressing the critical unmet needs of patients with relapsed SCLC.

##### Cardiovascular Disease Management

Inclisiran, approved in the U.S. and Europe in 2021, is the first cholesterol-lowering drug based on RNA interference (RNAi) technology.^414–432^ By reducing LDL-C levels with a twice-yearly subcutaneous injection, Inclisiran offers unparalleled convenience and efficacy, setting a new standard for lipid-lowering therapies.

##### Infectious Diseases

Approved in 2019, Pretomanid is a groundbreaking therapy for multidrug-resistant tuberculosis (MDR-TB). Its use in combination regimens significantly shortens treatment duration while improving efficacy, representing a crucial breakthrough in global TB management.^433–452^

#### Therapeutic biologics

##### Oncology

A standout global innovation in oncology is Tebentafusp (Kimmtrak), approved in 2022, which represents a paradigm shift in the treatment of uveal melanoma, a rare but aggressive cancer. Leveraging a novel T-cell receptor (TCR) platform, Tebentafusp targets Gp100 antigens expressed in melanoma cells. This marks a groundbreaking step in immunotherapy, addressing a patient population with historically poor outcomes and limited options, thereby setting a new standard for rare oncology indications.^453–472^

##### Rare Diseases

In rare diseases, Avalglucosidase Alfa (Nexviazyme), approved in 2021 for Pompe disease, exemplifies innovation in enzyme replacement therapy (ERT). This therapy enhances receptor binding and cellular uptake, significantly improving the treatment’s efficacy compared to earlier options.^473–491^ Avalglucosidase Alfa not only advances outcomes for patients with this lysosomal storage disorder but also underscores the continuous evolution of therapies targeting rare metabolic diseases.

##### Cardiovascular Diseases

Evinacumab, approved in 2021, is a first-in-class monoclonal antibody targeting ANGPTL3. This biologic is specifically indicated for patients with homozygous familial hypercholesterolemia (HoFH), a severe and rare genetic condition. By lowering LDL cholesterol levels independently of LDL receptors, Evinacumab provides a much-needed alternative for patients unresponsive to conventional therapies.^492–508^

##### Infectious Diseases

Approved in Europe in 2021, Regdanvimab is a monoclonal antibody therapy developed for the treatment of COVID-19. It has shown efficacy in preventing severe illness in high-risk patients, making it a critical addition to the global fight against the pandemic.

#### Cell and gene therapy products

Globally, the field of CGT has seen remarkable progress between 2019 and 2023, marking significant strides in addressing complex and rare diseases. Notable advancements include the approval of Zynteglo (betibeglogene autotemcel) in the United States in 2022 for transfusion-dependent beta-thalassemia (TDT). This gene therapy uses autologous hematopoietic stem cells modified with a functional beta-globin gene, offering a potentially curative option for patients.^509–516^ Similarly, Roctavian (valoctocogene roxaparvovec) was approved as the first licensed hemophilia A gene therapy that was conditionally approved in Europe in 2022 and approved in the United States in 2023, for utilizing an AAV5 vector to deliver a functional factor VIII gene, significantly reducing bleeding episodes in patients.^517–523^

In the domain of CAR-T cell therapies, Breyanzi (lisocabtagene maraleucel), approved in 2021, introduced a new generation of CAR-T therapies targeting CD19 for large B-cell lymphoma, offering enhanced efficacy and safety profiles compared to earlier products.^524–544^ Additionally, Abecma (idecabtagene vicleucel) was approved in 2021 as the first CAR-T cell therapy for relapsed or refractory multiple myeloma, addressing unmet needs in hematologic malignancies.^545–563^

#### Vaccine

Globally, significant strides have been made in vaccine development between 2019 and 2023, with groundbreaking advancements in both technology and target diseases. The Pfizer-BioNTech mRNA COVID-19 vaccine, approved in 2020, was the first mRNA-based vaccine to receive regulatory authorization, setting a new benchmark for vaccine technology. Its rapid development and high efficacy against SARS-CoV-2 represented a major scientific milestone.^564,565^ Similarly, Moderna’s mRNA vaccine (Spikevax), also authorized in 2020, expanded the use of mRNA technology, showcasing its scalability and adaptability to emerging viral threats.^564–574^ Beyond COVID-19, Mosquirix (RTS,S/AS01), approved by the WHO in 2021, became the first vaccine for malaria, providing critical protection against a disease responsible for significant global morbidity and mortality.^575–581^ This breakthrough in malaria prevention underscores the global commitment to addressing infectious diseases that disproportionately affect low-income regions.

### Comparison between China and globe in innovative drugs

#### Chemical drugs

China and global leaders have taken distinct approaches to chemical drug innovation. While China has made substantial progress in addressing unmet clinical needs in oncology and public health diseases, global markets, particularly the U.S. and Europe, maintain leadership in groundbreaking and platform-based therapies.

Targeting “Undruggable” Pathways: The U.S.‘s approval of Sotorasib^374–393^ for KRAS G12C mutations showcases its ability to tackle historically challenging drug targets. This marks a significant breakthrough in oncology, as KRAS mutations were long considered resistant to direct inhibition. In contrast, China’s advancements have predominantly focused on refining existing targets and expanding indications, such as through drugs like Surufatinib^225–235^ and Sunvozertinib.^236–245^ This strategy has led to the rapid development of next-generation inhibitors that improve upon existing treatments, addressing resistance and broadening patient eligibility.

The approval of Inclisiran, an RNAi-based therapy, highlights Western leadership in next-generation drug development platforms. In comparison, China is still in the early stages of adopting these technologies. Breakthrough therapies like Sotorasib and Pretomanid have leveraged extensive international multi-center trials to accelerate approval and global market entry. Conversely, China’s chemical drugs have primarily addressed domestic needs, with limited integration into international clinical research ecosystems.

China’s efforts in chemical drug innovation have demonstrated rapid progress, particularly in addressing domestic public health challenges and developing more accessible therapies. However, further advancements in cutting-edge technologies, global collaboration, and expansion into international markets will be pivotal for China to solidify its position as a global key player in pharmaceutical innovation.

#### Therapeutic biologics

China and the global landscape of innovative therapeutic biologics reveal both unique strengths and complementary priorities. Globally, significant strides have been made in addressing rare diseases, oncology, cardiovascular conditions, and infectious diseases through groundbreaking biologics such as Tebentafusp, Avalglucosidase Alfa, Evinacumab, and Regdanvimab. These innovations highlight a focus on precision medicine and meeting critical unmet needs in specific patient populations.

China, meanwhile, has demonstrated rapid progress in the development and approval of therapeutic biologics tailored to its unique healthcare landscape. Chinese innovations have increasingly focused on addressing high-burden diseases prevalent within the country, such as gastric and liver cancers, and expanding access to treatments for rare diseases. Examples include the approval of domestically developed biologics like Hunterase for Hunter syndrome and Risdiplam for spinal muscular atrophy. Additionally, China has prioritized biosimilars and collaborative efforts to enhance accessibility and affordability of biologics, aligning with its broader healthcare improvement goals.

The COVID-19 pandemic further underscored China’s capacity for rapid biologic innovation and large-scale manufacturing, as evidenced by the development and deployment of monoclonal antibodies like Etesevimab and JS016. These efforts complemented global advancements, such as Regdanvimab, in combating the pandemic.

In conclusion, while global efforts in therapeutic biologics often emphasize high-precision innovations targeting rare and complex conditions, China’s approach reflects a focus on broadening access, addressing local disease burdens, and building foundational capacities in biologic development. These complementary strategies collectively advance global healthcare innovation and improve patient outcomes worldwide.

#### Cell and gene therapy products

The regulatory landscape for CGT globally has also evolved to support these breakthroughs. Initiatives such as the FDA’s RMAT (Regenerative Medicine Advanced Therapy)^582–584^ designation and Europe’s PRIME (PRIority MEdicines) scheme have facilitated expedited review processes, promoting faster access to these life-changing therapies. These advancements reflect the growing international emphasis on fostering innovation in CGT.

China’s CGT sector has made impressive progress during the same period, with approvals for several domestically developed CAR-T therapies, including Relmacabtagene Autoleucel Injection, Inaticabtagene Autoleucel Injection, and Equecabtagene Autoleucel Injection, which target hematological malignancies. While these therapies align with global trends in CAR-T innovation, China’s advancements are distinguished by the rapid pace of regulatory approval under priority review pathways, such as the NMPA’s Conditional Approval mechanism.

However, when compared to global CGT leaders like the United States and Europe, China’s CGT field faces challenges in international market penetration and harmonization with global regulatory standards. For instance, while globally approved therapies like Zynteglo and Roctavian have expanded into international markets, China’s CAR-T therapies remain primarily focused on domestic needs, with limited international collaboration or adoption.

China has established a robust CGT pipeline, achieving significant milestones in regulatory efficiency and clinical implementation. However, global leaders have set benchmarks in the areas of market penetration, international collaboration, and diversification of CGT applications beyond hematological malignancies. Bridging these gaps will require China to enhance global regulatory harmonization, expand partnerships, and focus on CGT therapies addressing broader indications. Nonetheless, China’s rapid progress in CGT demonstrates its potential to become a key global player in this transformative field.

#### Vaccine

Compared to global leaders, China has focused on establishing a diverse portfolio of vaccines leveraging established platforms like inactivated and adenoviral vector vaccines. While mRNA platforms remain underexplored in China, its achievements in large-scale production, rapid approval processes, and supply to multiple countries have positioned it as a key contributor to global vaccine equity. The comparative advantage of China lies in its robust manufacturing capabilities and rapid regulatory pathways, while areas such as adopting cutting-edge platforms like mRNA and multi-valent vaccine formulations offer room for further growth. Together, these global and Chinese advancements underscore the importance of complementary strategies to strengthen vaccine innovation and accessibility worldwide.

Both the FDA and the EMA used Emergency Use Authorizations (EUAs) to rapidly approve COVID-19 vaccines.^585,586^ The FDA issued EUAs for the Pfizer-BioNTech and Moderna mRNA vaccines in December 2020, while the EMA followed with approvals shortly after. These mechanisms were put in place to speed up the approval process during the COVID-19 emergency, allowing the vaccines to be rolled out quickly despite some remaining uncertainties regarding long-term efficacy and safety. Both agencies used rolling reviews, where data from ongoing clinical trials were reviewed in stages, which helped expedite the decision-making process. In contrast, the NMPA also took steps to expedite the approval of COVID-19 vaccines in China, granting emergency use authorization for several vaccines. While NMPA also implemented expedited approval procedures for COVID-19 vaccines, including emergency use authorizations, the overall approval timeline may have been slightly longer compared to the FDA and EMA. However, the differences in approval times were often influenced by factors such as the availability of domestic data, regulatory requirements, and safety monitoring considerations.

## The promotion of drug innovation

### Regulatory support for innovative drug development in China

Following the discussion of individual disease areas, we now turn to the broader context of the promotion of innovative drug development and regulatory support, which provides essential context for understanding the environment in which these innovations are being developed. Regulatory support refers to the framework of policies, guidance, and resources provided by regulatory authorities to facilitate the efficient development, approval, and commercialization of pharmaceutical products, ensuring compliance with safety, efficacy, and quality standards.

In recent years, regulatory science research has been promoted by drug regulatory authorities in the U.S., the EU, Japan, China, and other countries, developing into a key strategic frontier discipline of the 21st century for global food and drug regulatory agencies. It has become an important battleground for influencing the next generation of the pharmaceutical industry on the international stage. While technological advancements in the 20^th^ century have addressed issues such as poverty, disease, and disability, they have also introduced new risks, exemplified by events like the 1937 Sulfanilamide disaster in the U. S., where a toxic solvent was used in the formulation of a liquid medicine, resulting in the deaths of over 100 people, including many children.^587,588^ This tragedy highlighted the dire need for stricter drug safety regulations and served as a turning point in the development of modern regulatory frameworks.

The Sulfanilamide disaster directly led to the enactment of the 1938 Federal Food, Drug, and Cosmetic Act, which granted the U.S. FDA the authority to ensure the safety of drugs before they could be marketed. This marked a major milestone in the evolution of the FDA and modern drug regulation. The FDA’s origins, however, trace back earlier to the 1906 Pure Food and Drug Act, the first federal law to regulate food and drugs in the United States, introduced to address widespread concerns about unsafe products and false claims. Together, these milestones established the FDA as a cornerstone of public health protection, laying the foundation for the emergence of regulatory science as a discipline.

Modern society faces the challenges of managing risks brought by emerging technologies, necessitating new regulatory tools, methods, and models—thus marking the emergence of the concept of “regulatory science.” Although the idea of regulatory science was initially proposed by public policy scholars, it was not widely accepted for a long time. In 1970, Alan Moghissi from the U.S. Environmental Protection Agency (EPA) first introduced the term “regulatory science” to describe the science used by the agency to formulate regulations. Alan Moghissi from the U.S. EPA first introduced the term “regulatory science“^589^ Since then, it has evolved through various interdisciplinary developments such as super-science, cross-science, evidence-based medicine, translational medicine, and precision medicine.^590–592^ Today, regulatory science has gained significant attention from major drug regulatory agencies worldwide and has become a strategic frontier discipline of the 21st century.

Based on China’s regulatory science trajectory and key milestones, we divide the scientific development of Chinese drug regulation into four stages:1) Initial Stage (Application of drug analysis technology, 1949–1984); 2) Development Stage (Establishment of drug registration standards, 1985–2014); 3) Acceleration Stage (Introduction of regulatory science concepts, 2015–2018); 4) Leapfrog Stage (Regulatory Science Action Plan, 2019–2023). In April 2019, the NMPA issued the “Notice on the Implementation of China’s Drug Regulatory Science Action Plan,” formally launching regulatory science research. Two batches of 19 key regulatory science projects were organized and implemented (Table S3 and S4). Additionally, 14 regulatory science research bases were established, and a total of 337 new regulatory tools, standards, and methods related to drug regulation were developed, providing important technical support for improving the quality and efficiency of China’s drug regulation. Notably, the rise and development of TCM regulatory science have accelerated the creation of an excellent regulatory system for TCM (TCMERS), which is tailored to the characteristics of TCM, rooted in Chinese distinctiveness, and globally advanced.^593–596^

In July 2023, the NMPA issued the “ Implementation Plan for Comprehensive Strengthening of the Drug Regulatory Science System,” emphasizing the strategic, forward-looking, and systematic development of the regulatory science system. It set new goals for the construction of the drug regulatory science system during the 14^th^ Five-Year Plan, focusing on pivotal tasks such as “strengthening and upgrading the construction of regulatory science infrastructure platforms,” “improving the regulatory science service support system,” and “breaking through key core technology bottlenecks in critical areas.” Through a series of innovations in regulatory tools, standards, and methods, the plan aims to foster high-level research and efficient translation in drug regulatory science, marking China’s entry into a new phase of comprehensive enhancement in the construction of its drug regulatory science system.^42,43^

### Regulatory measures to promote innovative drugs

In recent years, China’s biopharmaceutical industry has entered a period of rapid innovation and development, supported by favorable policies. Government agencies at various levels have promoted innovation accelerations across multiple stages, including R&D, clinical trials, market access, intellectual property, and medical insurance.^597^

#### Implementation of the marketing authorization holder (MAH) regulation

In 2015, China launched a pilot program for the MAH Regulation. The system was officially included in the revised *Drug Administration Law* in 2019, empowering innovative entities by allowing them to focus on their core strengths in R&D and manufacturing.

#### Implementation of International Council for Harmonisation (ICH) guidelines

In 2017, China acceded to the ICH and ascended to membership in the ICH Management Committee in 2018. The implementation of ICH guidelines in China has harmonized its drug registration standards with global benchmarks, reducing the time lag for approval of innovative drugs between China and the United States to near synchronization.

#### Clinical trial acceleration

The 2019 revision of the *Drug Administration Law* shifted the management of drug clinical trial institutions from an approval-based system to a record-based system, as outlined in the *Regulations on the Management of Drug Clinical Trial Institutions*. The transition altered the procedure from a system of explicit application approvals to one of consent, establishing a clear 60-working-day deadline, thereby expanding clinical supplies to accommodate the accommodate demand for drug R&D.

#### Establishment of drug development communication systems

In 2016, the NMPA issued the *Interim Measures for the Administration of Communication and Exchange in Drug Development and Technical Review*. In 2020, the *Drug Registration Regulation* integrated the communication mechanism into the basic drug registration system. Furthermore, in 2021, the *Implementation Opinions of the General Office of the State Council on Strengthening Drug Regulatory Capacity* increased the required frequency of communication meetings for innovative drugs. Through the improvement of the communication and exchange system, these measures aim to mitigate the risks inherent in innovative drug development while enhancing review efficiency and transparency.

#### Establishment of a patent compensation system

The 2017 *Opinions on Deepening the Reform of the Review and Approval System to Encourage Drug and Medical Device Innovation* introduced the patent term compensation system. In the 2021 revision of the *Patent Law*, this system was officially established to compensate for the time taken for the review and approval of new drug marketing. The compensation is capped at five years, with the total effective patent term not exceeding 14 years post-approval.

#### Optimization of the financing environment

In July 2019, China launched the Science and Technology Innovation Board (STAR Market), a new board of the stock exchange dedicated to fostering technological advancement and innovation. The biopharmaceutical industry is one of the pivotal sectors of the STAR Market, benefiting from improved liquidity, lower financing costs, and easier access to capital.

#### Normalization of medical insurance negotiations

In April 2021, the National Healthcare Security Administration (NHSA) and the National Health Commission issued the *Guiding Opinions on Establishing and Improving the ‘Dual-Channel’ Management Mechanism for Nationally Negotiated Drugs*. This mechanism ensures the supply, clinical use, and payment of negotiated drugs through two channels: designated medical institutions and designated retail pharmacies, with simultaneous integration into the medical insurance payment system. In September 2021, the NHSA and National Health Commission released the *Notice on Adapting to the Normalization of National Drug Negotiations and Continuously Promoting the Implementation of Negotiated Drugs*. This notice further emphasized the importance of normalizing drug negotiations and improved policies on drug allocation, procurement, and settlement. These measures facilitate the implementation of negotiated drugs and provide a policy window for the early market entry of domestically innovative drugs.

#### Comprehensive support for innovative drugs

In March 2024, the Government Work Report of the 14th National People’s Congress (NPC) highlighted “innovative drugs” as an emerging industry to be actively cultivated. In July 2024, the State Council approved the *Implementation Plan for Full-Chain Support for Innovative Drug Development*, providing comprehensive policy support in pricing, medical insurance, commercial insurance, drug allocation, financing, and optimizing review and approval processes.

#### Optimization of clinical trial approval

In July 2024, the NMPA issued the *Pilot Work Plan for Optimizing the Review and Approval Mechanism for Innovative Drug Clinical Trials*, aiming to further enhance the clinical trial review and approval process for innovative drugs. The plan strengthens the primary responsibility of drug clinical trial applicants and enhances the ability of stakeholders involved in clinical trials to identify and manage risks. Additionally, it seeks to establish systems and mechanisms that comprehensively improve the quality and efficiency of clinical trials. The goal is to complete the review and approval of innovative drug clinical trial applications within 30 working days, thereby reducing the time required to initiate clinical trials.^597^

#### Accelerated review and approval process for innovative drugs

##### Breakthrough Therapy Designation

**Fig. 5:**
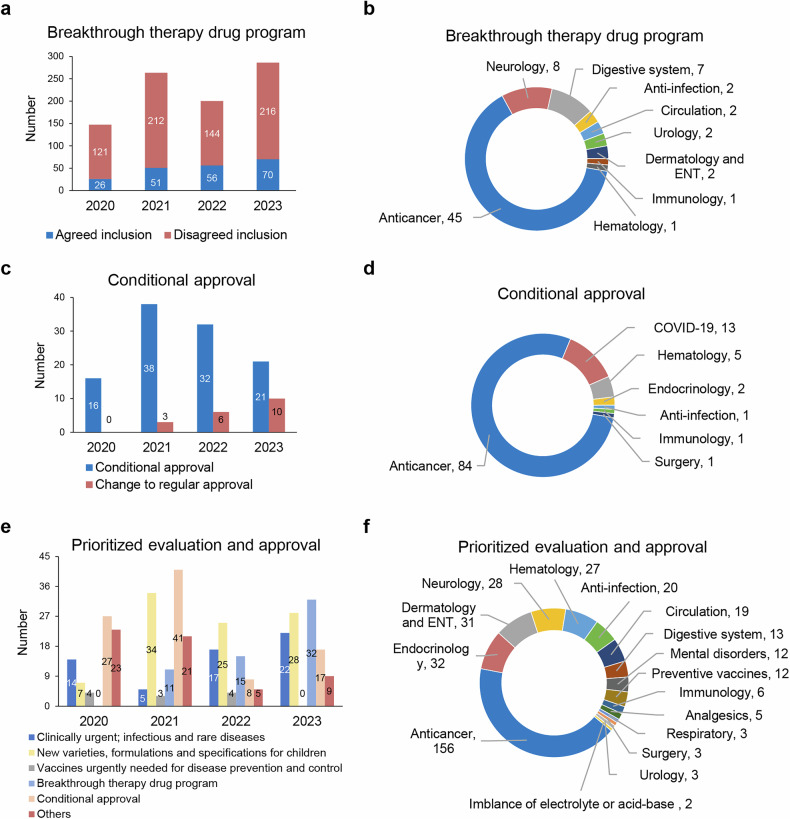
Overview of accelerated review and approval process for innovative drugs in China from 2020 to 2023. **a** Number of cases agreed or disagreed for inclusion under the Breakthrough Therapy program from 2020 to 2023. **b** Distribution of indication areas for drugs agreed for inclusion under Breakthrough Therapy program in the year of 2023. **c** Number of drugs conditionally approved and transitioned to regular approval from 2020 to 2023. **d** Distribution of indications for conditionally approved drugs from 2020 to 2023. **e** Number of drug registration applications under the Prioritized Evaluation and Approval procedure from 2020 to 2023. **f** Distribution of indications for drug registration applications under the Prioritized Evaluation and Approval procedure from 2020 to 2023.^95–99^

During clinical trials, innovative or modified drugs that are used for treating life-threatening or severely debilitating diseases, for which there are no effective treatments, or that show significant clinical advantages over existing treatments can apply for Breakthrough Therapy designation (BTD) in Phase I or II, usually no later than Phase III. In 2023, 286 applications were submitted for BTD, and 70 were granted, representing a 43% increase compared to 2022. The number of designations since the 2020 *Measures for the Administration of Drug Registration* was implemented is shown in Fig. 5a. Most of the designations were for oncology drugs (Fig. 5b).

##### Conditional Approval

Conditional approval can be granted for drugs that address life-threatening diseases lacking effective treatment, or in scenarios of public health emergencies when clinical trial data indicates effectiveness and predict clinical value. Since 2020, 95 drugs (107 indications) have been conditionally approved, with oncology drugs accounting for 79% (Fig. 5c, d). The conditional approval will be transitioned to full approval status upon the completion of post-marketing studies.

##### Prioritized Evaluation and Approval

Drugs with significant clinical value, including those treating rare diseases, pediatric diseases, major infectious diseases, and those listed under the BTD and Conditional Approval programs, are eligible for priority review. The review period for priority review is shortened to 130 days. In 2023, 108 applications (80 products) were accepted for priority review, an increase of 56.9% year-on-year (Fig. 5e). Oncology drugs accounted for the largest share of approvals (Fig. 5f).

### Breakthroughs in anti-cancer innovative drugs

In the realm of innovative drug development and regulation, anti-cancer drugs, as a key area addressing global health challenges, have received special attention and support. Given the high prevalence and complexity of cancer, the development of anti-cancer drugs faces significant technical challenges and unmet clinical needs. Therefore, regulatory agencies worldwide have implemented more refined and efficient regulatory measures specifically for anti-cancer drugs, in addition to promoting overall innovation in drug development. These measures have not only accelerated the market approval of anti-cancer drugs but also significantly improved their safety and efficacy, making anti-cancer drugs a strategic pioneer in the field of innovative medicines. They have provided patients with substantial health improvements and expanded treatment options.

The CDE of the NMPA has developed and published a series of guidelines to further refine the evaluation system for anti-cancer drugs. As of March 2024, the CDE had issued 503 technical guidelines, of which 61 were related to oncology indications. These guidelines have provided clear technical guidance and standards for pharmaceutical companies, playing a critical role in directing the development of innovative anti-cancer drugs, expediting the introduction of already-approved foreign anti-cancer drugs, and promoting the development and marketing of pediatric oncology drugs.

The 2020 *Measures for the Administration of Drug Registration* introduced four accelerated pathways: Conditional Approval, Breakthrough Therapy, Priority Review, and Special Approval. For diseases like cancer that pose a serious threat to public health, accelerating the development and approval of new drugs offers hope for survival to patients. As of March 2024, 70 anti-cancer drugs (covering 82 oncology indications) had been conditionally approved in China.^44,598^ The gradual establishment and refinement of China’s anti-cancer drug evaluation system have benefited from advancements in regulation, which have provided new tools and methods to support drug development.^599^

#### Single-arm trials offering treatment opportunities for patients with no alternatives

In 2020, the CDE issued two guidelines: the *Technical Guidelines for Communication Before Key Trials in Single-Arm Trials Supporting Anti-cancer Drug Marketing Approval* and the *Technical Guidelines for Communication Before Marketing Approval for Single-Arm Trials Supporting Anti-cancer Drug Applications*. In 2023, the CDE released the *Technical Guidelines for the Applicability of Single-Arm Clinical Trials for Supporting Anti-cancer Drug Applications*, systematically explaining the current scientific understanding of the use of single-arm trials for marketing applications. The first drug in China approved based on a single-arm trial was *Chidamide* (CS055), approved in 2014 for the treatment of relapsed and refractory peripheral T-cell lymphoma (PTCL).

#### Biomarkers enabling precision medicine

In 2021, the CDE published the *Technical Guidelines for the Use of Biomarkers in the Clinical Development of Anti-cancer Drugs*, detailing the definition and classification of biomarkers and emphasizing their application in evaluating the efficacy and safety of anti-cancer drugs.

#### Endpoints reflecting clinical benefit

The 2020 Measures for the Administration of Drug Registration and the accompanying Technical Guidelines for Conditional Approval stipulate that the selection of surrogate endpoints should be based on their ability to predict clinical benefit. For early-stage acute lymphoblastic leukemia (ALL) and acute myeloid leukemia (AML), the CDE issued several guidelines, including the Technical Guidelines for the Detection of Minimal Residual Disease (MRD) in Clinical Trials for Acute Lymphoblastic Leukemia, the Technical Guidelines for the Detection of MRD in Chronic Myeloid Leukemia (CML) Clinical Trials, and the Technical Guidelines for the Application of MRD in Multiple Myeloma (MM) Clinical Trials. When conducting clinical trials for cancer at different stages, considerations for trial endpoints may vary. The CDE issued the Technical Guidelines for Endpoints in Clinical Trials for Advanced NSCLC and the Technical Guidelines for Endpoints in Clinical Trials for Advanced Hepatocellular Carcinoma, detailing considerations for the use of surrogate endpoints at different stages of advanced cancer. According to the Guiding Principles for Anti-cancer Drug Development Based on Clinical Value, applicants are encouraged to use patient-reported outcomes (PRO) tools, such as quality of life and symptom assessments, to better understand how drugs alleviate symptoms and improve the quality of life for cancer patients.

The globalization of China’s anti-cancer drug approvals illustrates both progress and challenges. For example, Sintilimab, a PD-1 inhibitor approved in China, faced rejection by the FDA due to its reliance on data from the ORIENT-11 trial conducted exclusively in China. The FDA raised concerns about the homogeneity of the trial population and the comparator arm, which did not align with U.S. medical standards at the time.^600,601^ While the trial met its progression-free survival endpoint, the lack of diverse, multiregional clinical trial data limited its applicability to a global population. This case highlights the importance of conducting multiregional clinical trials to ensure Chinese-developed therapies meet international regulatory requirements and gain broader global acceptance.^602^ It is also important to note that other China-developed anti-PD-1 inhibitors, such as tislelizumab, have now been approved in the U.S. Tislelizumab, marketed as TEVIMBRA, was approved by the FDA for the first-line treatment of advanced or metastatic HER2-negative gastric or gastroesophageal junction adenocarcinoma in combination with chemotherapy for patients whose tumors express PD-L1 (≥1). This approval, based on the positive results of the global RATIONALE-305 Phase 3 trial, marks a significant step in BeiGene’s mission to provide transformative therapies for cancer patients globally.^603^ The trial demonstrated a 20% reduction in the risk of death, highlighting the international acceptance and potential for Chinese-developed cancer therapies like tislelizumab to succeed in global markets.

Chinese biotechnology companies have increasingly focused on oncology, particularly on immune checkpoint inhibitors (ICPIs), which have made significant contributions both in China and globally. In recent years, ICPIs have emerged as a key area of innovation, with several domestically developed ICPIs achieving regulatory approval and demonstrating clinical efficacy. Between 2005 and 2020, China approved 78 cancer drugs, including a growing number of ICPIs, with nearly half demonstrating overall survival (OS) benefits.^604,605^ Supported by faster regulatory pathways emphasizing clinical value, Chinese ICPIs are playing an increasingly important role in global oncology drug development, showcasing the country’s growing influence in this field.

### Global perspectives on regulatory support for innovative drug development

Globally, the evolution of regulatory frameworks has been pivotal in fostering innovative drug development, with agencies in the United States, Europe, and Japan leading efforts to streamline drug approval processes, integrate new technologies, and address unmet medical needs. The promotion of regulatory in these regions reflects their commitment to modernizing oversight systems and accelerating innovation in the pharmaceutical industry.

The U.S. FDA has long been at the forefront of regulatory innovation.^606^ Programs like the BTD,^122,123^ Accelerated Approval,^8,11,127,130,131,194^ and Priority Review^8,91^ have substantially reduced the time-to-market for novel therapeutics. As of 2023, the FDA approved over 100 drugs under the BTD program, with a strong focus on oncology, rare diseases, and gene therapies. The implementation of real-world evidence (RWE) in drug evaluations and the expansion of biomarkers for precision medicine have further enhanced the FDA’s ability to support cutting-edge therapies.

In Europe, the EMA has advanced regulatory innovation through its PRIME scheme and Adaptive Pathways program,^86,88,89,607^ aimed at accelerating the approval of drugs that address significant clinical gaps. The EMA’s emphasis on harmonizing regulations across member states has facilitated multi-country trials and improved access to innovative medicines.

Japan has embraced unique regulatory mechanisms, such as the Sakigake (Pioneering) designation,^608–613^ to incentivize domestic innovation and expedite the approval of breakthrough drugs. The use of conditional early approval pathways for regenerative medicine products has been instrumental in advancing therapies. Additionally, Japan’s focus on regulation has led to the establishment of innovative frameworks for real-world evidence and post-marketing surveillance.

International collaboration has emerged as a cornerstone of regulatory progress. The ICH has played a crucial role in aligning regulatory standards across major markets, enabling the global simultaneous development and approval of innovative drugs. Efforts like Project Orbis, spearheaded by the FDA, facilitate concurrent drug reviews among international regulators, significantly reducing delays in patient access to breakthrough therapies.

China’s drug regulatory system is increasingly aligning with global practices, and its future may include participation in international frameworks like Project Orbis,^614^ an FDA-led initiative enabling simultaneous submission and review of cancer drugs across multiple countries. Recent discussions between the U.S. and China on streamlining oncology drug development highlight this possibility. If China joins such initiatives, it could significantly enhance global collaboration, reduce approval timelines, and improve access to innovative therapies for patients worldwide. As regulatory improvements continue, China’s integration into global networks could further solidify its role as a key player in the international pharmaceutical landscape.

While China’s regulatory system has advanced rapidly, global agencies offer unique lessons in leveraging harmonization, adaptive pathways, and real-world data. For instance, the widespread use of biomarkers in the FDA’s precision medicine approvals and the EMA’s leadership in multi-country trials underscore the importance of integrating scientific advancements into regulatory processes. Moreover, global regulatory bodies have placed greater emphasis on post-marketing requirements and real-world evidence, areas where China’s system could further evolve to ensure long-term efficacy and safety. Globally, regulatory agencies continue to adapt to emerging scientific and societal challenges, setting benchmarks for efficiency, transparency, and innovation. By studying these global practices, China can refine its own regulatory framework, fostering a collaborative environment that accelerates drug development and enhances global access to life-saving therapies. The integration of international best practices, coupled with China’s own advancements, could significantly bolster global pharmaceutical innovation.

## China’s emerging role in global pharmaceutical innovation

China’s pharmaceutical industry has made significant strides in innovative drug development, with an increasing number of domestically developed drugs gaining approval and entering both domestic and international markets. Since 2021 to late 2024, Chinese authorities have approved 113 innovative drugs, contributing to a market scale of ~100 billion yuan (about 13.89 billion U.S. dollars).^615^

In comparison to global progress, China’s share of the innovative drug market remains relatively modest. In 2021, the United States accounted for over half of global innovative drug sales, Europe held 16 percent, while China stood at merely 3 percent.^616^ Despite this, Chinese pharmaceutical companies are actively expanding their presence in overseas markets. Collaborations with foreign firms and participation in global clinical trials have facilitated the entry of Chinese innovative drugs into international markets, including the United States and Europe. For instance, partnerships and licensing agreements have enabled Chinese-developed drugs to reach a broader patient population globally.

While China has made notable progress in innovative drug development, there remains significant potential for growth in both domestic and international markets. Continued investment in research and development, along with strategic collaborations, will be crucial for China to enhance its position in the global pharmaceutical landscape.

The improvements in research progress, infrastructure including research facilities, laboratories, and manufacturing capabilities, ecosystems such as the collaborative network of government, regulatory bodies, pharmaceutical companies, and academic institutions, and regulatory changes have propelled China’s innovative drug development. Chinese pharmaceutical companies have accelerated their integration into global drug development by conducting clinical trials abroad. Since July 2021, eight original innovative drugs from China have entered international markets, with seven of them being licensed to international partners. In 2024, 465 products under development reached the same development stage domestically and abroad, indicating a trend toward global synchronous development. Additionally, among the 2,623 products that entered clinical development since July 2021, the proportion of me-too products dropped to 35%, compared to 50% in 2021, reflecting the shift in China’s pharmaceutical industry toward drugs with higher clinical value potential.

In October 2023, Junshi Biosciences’ *Toripalimab* (marketed as LOQTORZI™ in the U.S.) received FDA approval, becoming the first and only FDA-approved drug for the treatment of nasopharyngeal carcinoma.^617–626^ The approval was supported by robust clinical trial data, including studies conducted in China and internationally, demonstrating its efficacy and safety. Junshi’s collaboration with international partners also played a crucial role in facilitating this approval.

In March 2024, BeiGene’s PD-1 product *Tislelizumab* (marketed as Baize’an or TEVIMBRA) was approved by the FDA as a monotherapy for adult patients with unresectable or metastatic esophageal squamous cell carcinoma (ESCC) who had previously undergone systemic chemotherapy (excluding PD-1/L1 inhibitors).^627–636^ In July 2024, CStone Pharmaceuticals’ *Sugemalimab* (marketed as Cejemly®) was approved by the European Commission for first-line treatment in combination with platinum-based chemotherapy for adult patients with metastatic non-small cell lung cancer without *EGFR* mutations or *ALK, ROS1*, or *RET* genomic alterations.^637–642^ In June 2024, Chinese biopharmaceutical company Hutchmed’s *c-MET* kinase inhibitor *Glumetinib* (SCC244) was approved by Japan’s Ministry of Health, Labor, and Welfare for the treatment of patients with METex14 skipping mutations in locally advanced or metastatic non-small cell lung cancer.^643^

**Fig. 6:**
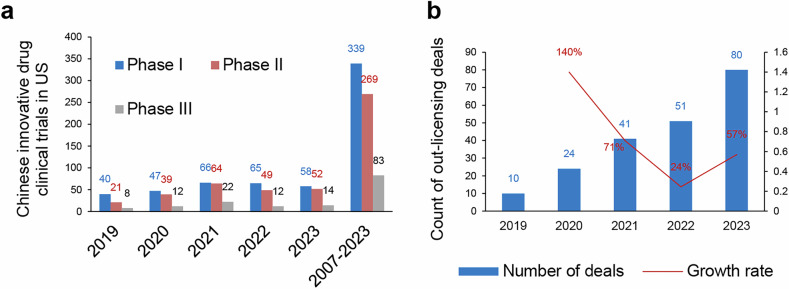
**a** Clinical trials of Chinese innovative drugs conducted in the U.S. from 2007 to 2023. **b** Number and growth rate of out-licensing deals of Chinese innovative drugs.^645^

An analysis of Chinese innovative drugs undergoing clinical trials in the U.S. from 2007 to 2023 indicates that China is evolving from a major pharmaceutical producer to an increasing participant in global drug development.^644^ Between 2007 and 2023, 177 Chinese pharmaceutical companies conducted 691 clinical trials for 350 original innovative drugs, targeting 499 indications in the U.S., including 399 Phase I trials (49%), 269 Phase II trials (39%), and 83 Phase III trials (12%)(Fig. 6a).^644^ Six Chinese innovative drugs have been approved for marketing in the U.S., covering 10 indications, including BeiGene’s *Zanubrutinib* (2019), Legend Biotech’s *Cilta-cel* (2022), Ascletis’ *Benvitimod* (2022), Junshi Biosciences’ *Toripalimab* (2023), Hutchmed’s *Fruquintinib* (2023), and Elpiscience’s *Agarcetin-α* (2023). Of the 350 original innovative drugs, 182 (52%) were NMEs, 162 (46%) were new biologics, and six (2%) were traditional Chinese medicines. Small molecule drugs and monoclonal antibodies accounted for 45% and 22%, respectively; bispecific antibodies for 8%, ADC for 7%, and cell and gene therapies for 5%.

Oncology remains the primary focus of Chinese pharmaceutical companies‘ drug development. Of the 691 clinical trials conducted, 358 trials (72%) involved 230 oncology drugs. The top five targets for anti-cancer drugs were PD-1, EGFR, PD-L1, HER2, and Claudin 18.2. Most of the clinical trials for Chinese innovative drugs in the U.S. (82%) were initiated between 2019 and 2023, reflecting a rapid growth.^114–117^

As reported by Qianwei Ge et al., between 2007 and 2023, 311 clinical stage transitions occurred for 75 drug development projects.^644^ The success rate for Phase II trials was 17%, much lower than 41% for Phase I and 36% for Phase III trials. The probability of approval after submission to regulatory agencies was 67%. The success rate for Phase I clinical trials of new biologics was much lower than for new molecular entities (16% vs. 49%), but new biologics had a higher success rate in Phase III (50% vs. 31%).^644^

Over the past five years, China’s innovative drug out-licensing has made significant progress, showing several key trends (Fig. 6b). 1) There has been a notable growth in the number of transactions: in 2023, the number of out-licensing deals for innovative drugs reached 80, a 57% increase compared to 51 deals in 2022, reflecting the increasing international recognition of Chinese innovative drugs.^645^ 2) The transaction value and structure have been optimized: in 2023, the total transaction value for out-licensing exceeded $46.5 billion, a 69% increase from $27.55 billion in 2022, with a higher proportion of upfront payments indicating growing optimism among international partners about the prospects of Chinese innovative drugs.^646^ 3) The concentration of deals remains in key sectors such as oncology and immunotherapy, which have bolstered the global influence of Chinese pharmaceutical companies, particularly in the development of innovative therapies targeting cancer and immune-related diseases. 4) Geographically, while Europe and North America remain primary markets for out-licensing, emerging markets in Southeast Asia and Latin America are gaining traction, providing new growth opportunities and furthering the global reach of Chinese innovative drugs.

The Chinese government has encouraged pharmaceutical companies to globalize, especially in innovative drug development. Although the NMPA does not directly manage the process of Chinese innovative drugs entering overseas markets, such as marketing, sales, or approvals, its regulatory activities and policies have significant indirect effects. These effects include setting regulatory standards, guiding drug development practices, facilitating international collaborations, and providing incentives for innovation, which collectively influence the global competitiveness and market access of Chinese drugs. Additionally, the Chinese government has provided ongoing support for the globalization of pharmaceutical companies, particularly through policies that encourage innovation and international expansion.

## Conclusion and future prospective

The landscape of innovative drug development is driven by several critical clinical needs, many of which remain unmet despite significant scientific advancements. These clinical needs span a wide range of conditions, from rare and orphan diseases to the more common but complex challenges of cancer, cardiovascular diseases, and neurodegenerative disorders. The increasing prevalence of chronic diseases such as diabetes and obesity, as well as the rise of antimicrobial resistance (AMR), underscores the urgent need for new, effective therapies.

In oncology, for example, while there has been significant progress in immunotherapy and targeted therapies, many cancers still have limited treatment options, especially for advanced stages and metastatic diseases. The clinical need for therapies that can address drug resistance, improve patient survival rates, and reduce side effects is particularly pressing. Similarly, in the field of neurodegenerative diseases, such as Alzheimer’s and Parkinson’s, there is an overwhelming demand for drugs that can modify disease progression, not just alleviate symptoms. Despite substantial investment in research, these diseases remain largely untreatable, highlighting a significant gap in the development of effective therapeutic options.

In addition to these complex conditions, emerging health threats such as the global COVID-19 pandemic have exposed vulnerabilities in existing drug development systems.^354^ The urgent need for rapid vaccine and antiviral drug development during the pandemic has underscored the importance of adaptive regulatory frameworks, international collaboration, and the ability to swiftly mobilize clinical trials. The pandemic has also highlighted the need for innovation in vaccine technology, diagnostics, and treatment options that can be rapidly deployed in response to global health crises.

Moreover, the rise of personalized medicine is transforming drug development, as there is a growing need for therapies that are tailored to individual patient profiles. Precision medicine aims to customize healthcare, with the goal of delivering more effective treatments based on genetic, environmental, and lifestyle factors. This approach requires advanced diagnostic tools and an understanding of the molecular mechanisms underlying diseases, pushing the boundaries of regulatory frameworks to accommodate new methods of treatment and testing.

Addressing these clinical needs requires not only innovative scientific research but also a regulatory system that can support the accelerated development and approval of novel therapies. The integration of regulatory support mechanisms that facilitate adaptive clinical trial designs, expedite the approval process for high-need drugs, and promote international cooperation is critical to meeting these challenges. Ensuring that new treatments can be efficiently brought to market is essential to improving patient outcomes and addressing the ongoing gaps in global healthcare.

The global innovative drug development has seen unprecedented advancements over the past decade, driven by cutting-edge technologies, diversified funding sources, and evolving regulatory frameworks. The success of novel platforms such as mRNA technology, CRISPR gene editing, and bispecific antibodies has redefined therapeutic possibilities and accelerated breakthroughs in areas like oncology, rare diseases, and infectious diseases. Nations like the United States and Europe have maintained leadership through robust collaboration between academia, government, and industry, while emerging markets like China are rapidly closing the gap. However, global challenges persist, including high R&D costs, disparities in access to innovation, and the need for harmonized regulatory standards. Strengthening global partnerships and leveraging complementary strengths will be essential to overcoming these challenges, fostering equitable access, and addressing unmet clinical needs across diverse populations. By aligning efforts internationally, the pharmaceutical sector can continue to deliver transformative therapies that improve health outcomes worldwide.

Innovative drug development is a complex and long-term process influenced by various factors such as technology, market demand, regulatory policies, and funding. Companies and research institutions need to closely monitor these trends and continuously adjust their strategies to address future challenges and opportunities in drug development. The pivotal role of collaboration between governmental bodies, academic institutions, and the private sector will be indispensable for propelling innovative drug development forward in China.

The key opportunities for innovative drug development in China encompass the following aspects:

1. Macroeconomic policy:
  The recently enacted Implementation Plan for Full-Chain Support for Innovative Drug Development will continue to foster innovation in China’s pharmaceutical sector. Future policies are expected to accelerate development by streamlining the drug review and approval process, enhancing intellectual property protection, and offering tax incentives. These policies aim to reduce approval timelines through expedited pathways and rolling reviews, particularly for critical therapies with high unmet medical needs. Intellectual property laws will be strengthened to encourage innovation, with specific incentives for high-tech areas like gene therapy and biologics. Additionally, tax relief will be provided for companies engaged in R&D, including deductions for research expenses. These policies also focus on enhancing clinical trial efficiency by reducing regulatory barriers and promoting international multi-center trials, ensuring that China’s drug development process is globally competitive and aligned with international standards.
2. Technological Innovation:
  The integration of emerging technologies such as biotechnology, gene editing, and artificial intelligence into drug development is already improving efficiency and success rates. China’s advancements in CGT, protein degraders, and covalent drugs highlight the country’s commitment to adopting and advancing novel paradigms. This progress is aligned with global innovation trends and positions China as a key player in shaping the future of drug development.
3. Clinical demand:
  China’s vast and diverse population, combined with a broad spectrum of diseases, provides significant clinical resources and market demand for innovative drugs. Areas such as oncology, rare diseases, and chronic illnesses are particularly critical, as unmet medical needs in these fields drive much of the current R&D activity. This clinical demand not only supports drug development but also provides a real-world testing ground for innovative therapies.
4. R&D investment:
  Increasing attention from both the capital markets and government support is expected to lead to greater investment in drug development. The growing availability of diverse financing channels ensures that sufficient funding is directed toward high-risk, high-reward R&D projects, fostering a robust drug development ecosystem in China.
5. Regulatory science:
  The exploration and application of regulatory science have significantly improved China’s regulatory environment. Efforts to refine drug evaluation processes, align with international standards, and accelerate the approval of new therapies are enhancing China’s position as a global hub for drug development.
6. International collaboration:

As China’s pharmaceutical sector aligns more closely with international standards, opportunities for global partnerships and joint ventures continue to expand. These collaborations not only provide access to international markets but also enable Chinese companies to contribute to and learn from global innovation efforts, accelerating the pace of drug development.

In summary, driven by macroeconomic policies and regulatory promotion, China’s innovative drug development is poised for significant advancements in small molecules, biologics, and traditional Chinese medicines. Strengthening regulation, expediting the review process, and deepening international cooperation will position China as a major player in global pharmaceutical innovation, contributing to the enhancement of human health and well-being.

## Supplementary information

Supplementary Information
