# Supplementary material for: Current landscape of innovative drug development and regulatory support in China
Source: Signal Transduct Target Ther. 2025 Jul 22;10:220. doi: 10.1038/s41392-025-02267-y (PMC12280122; doi:10.1038/s41392-025-02267-y)
Supplement: Supplementary file 1 — Supplementary Information [file 41392_2025_2267_MOESM1_ESM.docx]

Supplementary Materials for

**Current landscape of innovative drug development and regulatory support in China**

**Ruirong Tan, Hua Hua, Siyuan Zhou, Zhimin Yang****, Changming Yang, Guo Huang, Jin Zeng, Junning Zhao**

Correspondence to: zarmy@189.cn

**This PDF file includes:**

Supplementary Text

Tables S1 to S4

Supplementary Text

The unique role of TCM in pharmaceutical innovation in China

Traditional Chinese Medicine (TCM) occupies a singular position in the global pharmaceutical landscape, combining centuries-old medical wisdom with modern scientific advancements. Unlike conventional drug development, TCM innovation is rooted in a distinctive "disease-syndrome combination" research paradigm, which integrates traditional diagnostic principles with contemporary clinical methodologies. This approach highlights the unique capability of TCM to address complex and multifaceted health conditions, offering holistic and personalized therapeutic options that set it apart from mainstream pharmaceutical practices.

In China, the number of innovative traditional Chinese medicines (TCMs) has steadily increased. Many of the approved innovative TCMs have adopted a "disease-syndrome combination" research and development model and are approved for indications that align with the clinical advantages and characteristics of TCM, including diabetic retinopathy, diabetic nephropathy, knee osteoarthritis, insomnia, seasonal allergic rhinitis, menopausal syndrome, aphthous ulcers, chronic non-atrophic gastritis, functional dyspepsia, pediatric tic disorder, and acute pediatric bronchitis, providing more treatment options for patients with these conditions.

Over the past five years, the number of innovative TCMs approved for marketing has steadily increased. Many of the approved innovative TCM products adopt a "disease-syndrome combination" research model and are approved for indications that align with the clinical advantages of TCM and the characteristics of traditional medicine. These indications include diabetic retinopathy, diabetic nephropathy, knee osteoarthritis, insomnia, seasonal allergic rhinitis, menopausal syndrome, aphthous ulcers, chronic non-atrophic gastritis, functional dyspepsia, and more, providing patients with more treatment options for these conditions.

*Ramulus Mori (Sangzhi) Alkaloids Tablets*, whose main ingredient is derived from mulberry branch total alkaloids, was the first new anti-diabetic TCM approved in the past decade. It is indicated for use in conjunction with diet control and exercise to treat type 2 diabetes. This product effectively reduces glycated hemoglobin levels in patients with type 2 diabetes, offering a new treatment option for these patients.^1-5^

*Icariin Soft Capsules* are used to treat unresectable hepatocellular carcinoma in patients who are either unsuitable for or refuse standard treatment and have not previously received systemic therapy. Patients must meet at least two of the following peripheral blood biomarker criteria: AFP ≥ 400 ng/mL; TNF-α < 2.5 pg/mL; IFN-γ ≥ 7.0 pg/mL. This product, derived from the TCM herb *Epimedium*, is a Category 1.2 innovative TCM and provides a new treatment option for patients with hepatocellular carcinoma.^6-11^

*Yiqi Tongqiao Pills*, which invigorate *qi* and dispel *wind*, are used to treat seasonal allergic rhinitis in patients with lung-spleen *qi* deficiency syndrome. This product is a TCM compound preparation made from 14 medicinal ingredients, including *Astragalus* and *Saposhnikovia*, and was developed based on clinical experience. Randomized, double-blind, placebo-controlled, multi-center clinical trials have been conducted, and the product provides a new treatment option for patients with seasonal allergic rhinitis.

The approval and marketing of these innovative TCMs not only demonstrate the modernization of TCM research but also provide new treatment options for patients with these conditions, promoting the development of China's pharmaceutical innovation capabilities and meeting clinical needs. The deepening changes of the national drug review and approval system has improved the efficiency of TCM innovation and accelerated the time to market for innovative TCMs, offering patients more treatment options while advancing the modernization of TCM and fostering the rapid development of innovative TCM products.

The distinctiveness of TCM lies in its integration of traditional therapeutic principles with modern scientific rigor, creating innovative pathways to address unmet medical needs. With robust regulatory changes and growing scientific support, TCM has not only modernized but also carved out a unique space within China's pharmaceutical innovation ecosystem. By emphasizing holistic treatment, personalized care, and multi-targeted mechanisms, TCM stands as a critical complement to conventional drug development, exemplifying a uniquely Chinese contribution to global healthcare challenges.

References

1. Liu, D., et al. Ramulus mori (Sangzhi) alkaloids regulates gut microbiota disorder and its metabolism profiles in obese mice induced by a high-fat diet. *Front. Pharmacol.* **14,** 1166635 (2023).

2. Chen, Y.-M., et al. Ramulus mori (sangzhi) alkaloids alleviate high-fat diet-induced obesity and nonalcoholic fatty liver disease in mice. *Antioxidants* **11,** 905 (2022).

3. Sun, Q.-W., et al. Ramulus mori (sangzhi) alkaloids ameliorate obesity-linked adipose tissue metabolism and inflammation in mice. *Nutrients* **14,** 5050 (2022).

4. Liu, Q., et al. Ramulus Mori (Sangzhi) alkaloids (SZ-A) ameliorate glucose metabolism accompanied by the modulation of gut microbiota and ileal inflammatory damage in type 2 diabetic KKAy mice. *Front. Pharmacol.* **12,** 642400 (2021).

5. An, X., et al. Ramulus Mori (Sangzhi) alkaloids tablets for diabetes mellitus: A regulatory perspective. *Fitoterapia* **166,** 105444 (2023).

6. Huong, N.T., Son, N.T. Icaritin: A phytomolecule with enormous pharmacological values. *Phytochemistry* 113772 (2023).

7. Yang, X.-J., Xi, Y.-M., Li, Z.-J. Icaritin: A novel natural candidate for hematological malignancies therapy. *BioMed research international* **2019,** 4860268 (2019).

8. Zhang, C., Sui, X., Jiang, Y., Wang, X., Wang, S. Antitumor effects of icaritin and the molecular mechanisms. *Discov. Med.* **29,** 5-16 (2020).

9. Bi, Z., Zhang, W., Yan, X. Anti-inflammatory and immunoregulatory effects of icariin and icaritin. *Biomed. Pharmacother.* **151,** 113180 (2022).

10. Gao, L., Zhang, S.-Q. Antiosteoporosis effects, pharmacokinetics, and drug delivery systems of icaritin: advances and prospects. *Pharmaceuticals* **15,** 397 (2022).

11. Huang, J., Yuan, L., Wang, X., Zhang, T.-L., Wang, K. Icaritin and its glycosides enhance osteoblastic, but suppress osteoclastic, differentiation and activity in vitro. *Life Sci.* **81,** 832-40 (2007).

Table S1.

List of category 1 innovative drugs approved in China between 2018 and 2023

| **No.** | **Approved Drug Name** | **Approval Number** | **Marketing Authorization Holder** | **Approval Date** | **Domestic/International** |
| --- | --- | --- | --- | --- | --- |
| 1 | Anlotinib Hydrochloride Capsules | Approval No. H20180002 | Chia Tai Tianqing Pharmaceutical Group Co., Ltd. | 05/14/2018 | Jiangsu, China |
| 2 | Pyrotinib Maleate Tablets | Approval No. H20180012 | Jiangsu Hengrui Medicine Co., Ltd. | 08/16/2018 | Jiangsu, China |
| 3 | Fruquintinib Capsules | Approval No. H20180015 | Hutchison MediPharma (Shanghai) Co., Ltd. | 09/05/2018 | Shanghai, China |
| 4 | Emicizumab Injection | Approval No. SJ20180025 | Roche Pharma (Schweiz) AG | 12/04/2018 | Switzerland |
| 5 | Toripalimab Injection | Approval No. S20180015 | Shanghai Junshi Biosciences Co., Ltd. | 12/17/2018 | Shanghai, China |
| 6 | Roxadustat Capsules | Approval No. H20180023 | FibroGen China Medical Technology Development Co., Ltd. | 12/18/2018 | Beijing, China |
| 7 | Jinrong Granules | Approval No. Z20180002 | Guangzhou Qiji Pharmaceutical Technology Co., Ltd. | 12/28/2018 | Guangdong, China |
| 8 | Sintilimab Injection | Approval No. S20180016 | Innovent Biologics (Suzhou) Co., Ltd. | 12/28/2018 | Jiangsu, China |
| 9 | Pegylated Exenatide Injection | Approval No. H20190024 | Jiangsu Hansoh Pharmaceutical Group Co., Ltd. | 05/05/2019 | Jiangsu, China |
| 10 | Benvitimod Cream | Approval No. H20190026 | Guangdong Zhonghao Pharmaceutical Co., Ltd. | 05/29/2019 | Guangdong, China |
| 11 | Sodium Oligomannate Capsules | Approval No. H20190031 (Conditional) | Green Valley (Shanghai) Pharmaceuticals Co., Ltd. | 11/02/2019 | Shanghai, China |
| 12 | Flumatinib Mesylate | Approval No. H20190032 | Jiangsu Hansoh Pharmaceutical Group Co., Ltd. | 11/22/2019 | Jiangsu, China |
| 13 | Remimazolam Tosylate for Injection | Approval No. H20190034 | Jiangsu Hengrui Medicine Co., Ltd. | 12/26/2019 | Jiangsu, China |
| 14 | Niraparib Tosylate Capsules | Approval No. H20190035 (Conditional) | Zai Lab (Shanghai) Co., Ltd. | 12/26/2019 | Shanghai, China |
| 15 | Chloroprene Hydrochloride Capsules | Approval No. H20200001 | Beijing Kangwei Biotech Co., Ltd. | 02/11/2020 | Beijing, China |
| 16 | Almonertinib Mesylate Tablets | Approval No. H20200004 | Jiangsu Hansoh Pharmaceutical Group Co., Ltd. | 03/17/2020 | Jiangsu, China |
| 17 | Quisibenzonium Bromide Nasal Spray | Approval No. H20200003 | Silver Valley Pharmaceutical Co., Ltd. | 03/17/2020 | Beijing, China |
| 18 | Siponimod Tablets | Registration Certificate No. H20200010 | Novartis Pharma Schweiz AG | 05/07/2020 | Switzerland |
| 19 | Zanubrutinib Capsules | Approval No. H20200005 | BeiGene (Suzhou) Biopharmaceutical Co., Ltd. | 06/02/2020 | Jiangsu, China |
| 20 | Ravidasvir Hydrochloride Tablets | Approval No. H20200008 | Ascletis Pharma (Hangzhou) Co., Ltd. | 07/29/2020 | Zhejiang, China |
| 21 | Ensartinib Hydrochloride Capsules | Approval No. H20200009 | Betta Pharmaceuticals Co., Ltd. | 11/17/2020 | Zhejiang, China |
| 22 | Fluzoparib Capsules | Approval No. H20200014 | Jiangsu Hengrui Medicine Co., Ltd. | 12/14/2020 | Jiangsu, China |
| 23 | Etomidate Phosphate Injection | Approval No. H20200013 | Liaoning Haisco Pharmaceutical Co., Ltd. | 12/11/2020 | Liaoning, China |
| 24 | Imidafenib Phosphate Capsules | Approval No. H20200015 | Yichang East Sunshine Changjiang Pharmaceutical Co., Ltd. | 12/21/2020 | Hubei, China |
| 25 | Orelabrutinib Tablets | Approval No. H20200016 | Beijing InnoCare Pharma Tech Co., Ltd. | 12/25/2020 | Beijing, China |
| 26 | Surufatinib Capsules | Approval No. H20200017 | Hutchison MediPharma (Shanghai) Co., Ltd. | 12/29/2020 | Shanghai, China |
| 27 | Furmonertinib Mesylate Tablets | Approval No. H20210008 (Conditional) | Astellas Pharmaceuticals Shanghai Co., Ltd. | 03/03/2021 | Shanghai, China |
| 28 | Taltirelin for Injection | Approval No. S20210008 | Rongchang Biopharmaceutical Co., Ltd. | 03/09/2021 | Shandong, China |
| 29 | Utidelone Injection | Approval No. H20210011 | Chengdu Huahao Zhongtian Pharmaceutical Co., Ltd. | 03/11/2021 | Sichuan, China |
| 30 | Pralsetinib Capsules | Approval No. HJ20210018 | Genentech Inc. (A Member of the Roche Group) | 03/23/2021 | USA |
| 31 | Pamiparib Capsules | Approval No. H20210016 (Conditional) | BeiGene (Suzhou) Biopharmaceutical Co., Ltd. | 04/30/2021 | Jiangsu, China |
| 32 | Cantharidin Tablets | Approval No. H20210019 | Shanghai Menox Pharmaceuticals Co., Ltd. | 06/01/2021 | Shanghai, China |
| 33 | Vedicitumab for Injection | Approval No. S20210017 (Conditional) | Rongchang Biopharmaceutical Co., Ltd. | 06/08/2021 | Shandong, China |
| 34 | Donafenib Tosylate Tablets | Approval No. H20210020 | Jiangsu Zejing Pharmaceutical Co., Ltd. | 06/09/2021 | Jiangsu, China |
| 35 | Risdiplam for Oral Solution Powder | Approval No. HJ20210045 | F. Hoffmann-La Roche Ltd. (Manufacturer) Roche Pharma (Schweiz) AG (MAH) | 06/16/2021 | Switzerland |
| 36 | Eltrombopag Ethanolamine Tablets | Approval No. H20210021 | Jiangsu Hengrui Medicine Co., Ltd. | 06/16/2021 | Jiangsu, China |
| 37 | Savolitinib Tablets | Approval No. H20210026 | Hutchison MediPharma (Shanghai) Co., Ltd. | 06/22/2021 | Shanghai, China |
| 38 | Adefovir Dipivoxil Tablets | Approval No. H20210029 | Jiangsu Hansoh Pharmaceutical Group Co., Ltd. | 06/22/2021 | Jiangsu, China |
| 39 | Ezetimibe Tablets | Approval No. H20210030 | Zhejiang Hisun Pharmaceutical Co., Ltd. | 06/25/2021 | Zhejiang, China |
| 40 | Elaprivirine Tablets | Approval No. H20210032 | Jiangsu Aidea Pharmaceutical Co., Ltd. | 06/25/2021 | Jiangsu, China |
| 41 | Azvudine Tablets | Approval No. H20210035 (Conditional) | Henan Genuine Biotech Co., Ltd. | 07/21/2021 | Henan, China |
| 42 | Yiqi Tongqiao Pills | Approval No. Z20210002 | Tianjin Oriental Huakang Pharmaceutical Technology Development Co., Ltd. (MAH: Yangzijiang Pharmaceutical Group Sichuan Hairong Pharmaceutical Co., Ltd.) | 09/13/2021 | Tianjin / Sichuan, China |
| 43 | Siglita Sodium Tablets | Approval No. H20210046 | Chengdu Chipscreen Biosciences Co., Ltd. | 10/22/2021 | Sichuan, China |
| 44 | Yinqiao Qingre Tablets | Approval No. Z20210003 | Jiangsu Kanion Pharmaceutical Co., Ltd. | 11/09/2021 | Jiangsu, China |
| 45 | Olverembatinib Tosylate Tablets | Approval No. H20210048 (Conditional) | Guangzhou Sun Gene Biopharmaceutical Co., Ltd. | 11/24/2021 | Guangdong, China |
| 46 | Envafolimab Injection | Approval No. S20210046 (Conditional) | Sichuan Sorrento Biopharmaceutical Co., Ltd. | 11/24/2021 | Sichuan, China |
| 47 | Xuanyi Jianbone Tablets | Approval No. Z20210004 | Hunan Fangsheng Pharmaceutical Co., Ltd. | 11/24/2021 | Hunan, China |
| 48 | Qizhi Yishen Capsules | Approval No. Z20210005 | Shandong Phoenix Pharmaceutical Co., Ltd. | 11/24/2021 | Shandong, China |
| 49 | Kunxin Ning Granules | Approval No. Z20210006 | Tasly Pharmaceutical Group Co., Ltd. | 11/24/2021 | Tianjin, China |
| 50 | Huzhen Qingfeng Capsules | Approval No. Z20210007 | Yili Pharmaceutical Co., Ltd. | 12/14/2021 | Guangdong, China |
| 51 | Jieyu Chufan Capsules | Approval No. Z20210008 | Yiling Pharmaceutical Co., Ltd. | 12/14/2021 | Hebei, China |
| 52 | Sugelimumab Injection | Approval No. S20210053 | Pfizer Investment Co., Ltd. | 12/20/2021 | Shanghai, China |
| 53 | Qirui Weishu Capsules | Approval No. Z20210009 | Jianmin Pharmaceutical Group Co., Ltd. | 12/31/2021 | Hubei, China |
| 54 | Proline Gagliflozin Tablets | Approval No. H20210052 | Jiangsu Hengrui Medicine Co., Ltd. | 12/31/2021 | Jiangsu, China |
| 55 | Dalpiciclib Hydroxyethylsulfonate Tablets | Approval No. H20210054 | Jiangsu Hengrui Medicine Co., Ltd. | 12/31/2021 | Jiangsu, China |
| 56 | Icaritin Soft Capsules | Approval No. Z20220002 (Conditional) | Beijing Shenogen Pharma Co., Ltd. | 01/10/2022 | Beijing, China |
| 57 | Ombacitinib Injection | Approval No. S20220003 | Huabei Pharmaceutical Group New Drug R&D Co., Ltd. | 01/25/2022 | Hebei, China |
| 58 | Abrocitinib Tablets | Approval No. HJ20220029 | Pfizer Inc. | 04/08/2022 | Germany |
| 59 | Vericiguat Tablets | Approval No. HJ20220050 | Bayer AG | 05/18/2022 | Germany |
| 60 | Finerenone Tablets | Approval No. HJ20220057 | Bayer AG | 06/28/2022 | Germany |
| 61 | Rivaroxaban Tablets | Approval No. H20220016 (Conditional) | Jiangsu Hengrui Medicine Co., Ltd. | 06/29/2022 | Jiangsu, China |
| 62 | Cadonilimab Injection | Approval No. S20220018 (Conditional) | Akeso Pharmaceuticals Co., Ltd. | 06/28/2022 | Guangdong, China |
| 63 | Guangjin Qian Grass Total Flavonoids Capsules | Approval No. Z20220003 | Wuhan Guanggu Renfu Biopharmaceutical Co., Ltd. | 09/14/2022 | Hubei, China |
| 64 | Dorzagliatin Tablets | Approval No. H20220024 | Hua Medicine (Shanghai) Co., Ltd. | 09/30/2022 | Shanghai, China |
| 65 | Toludivenlafaxine Hydrobromide Sustained-Release Tablets | Approval No. H20220028 | Shandong Luye Pharmaceutical Co., Ltd. | 11/01/2022 | Shandong, China |
| 66 | Limpride Tablets | Approval No. H20220030 (Conditional) | Shanghai Yingli Pharmaceuticals Co., Ltd. | 11/08/2022 | Shanghai, China |
| 67 | Pesotumab Injection | Approval No. SJ20220020 | Boehringer Ingelheim International GmbH | 12/13/2022 | Germany |
| 68 | Qijiao Tiaojing Granules | Approval No. Z20220007 | Hunan Anbang Pharmaceutical Co., Ltd. | 12/27/2022 | Hunan, China |
| 69 | Shengbu Capsule | Approval No. Z20220008 | Xinjiang Huachun Biological Pharmaceutical Co., Ltd. | 12/29/2022 | Xinjiang, China |
| 70 | Enovidine Tablets | Approval No. H20220033 | Jiangsu Aidea Pharmaceutical Co., Ltd. | 12/30/2022 | Jiangsu, China |
| 71 | Mobocertinib Succinate Capsules | Approval No. HJ20230001 (Conditional) | Takeda Pharmaceuticals USA, Inc. | 01/11/2023 | USA |
| 72 | Senoteravir Tablets/Ritonavir Tablets Combo Pack | Approval No. H20230001 (Conditional) | Hainan Simcere Pharmaceuticals Co., Ltd. | 01/28/2023 | Hainan, China |
| 73 | Deutromedetomidine Hydrobromide Tablets | Approval No. H20230002 (Conditional) | Shanghai Wanshi Biopharmaceutical Technology Co., Ltd. | 01/28/2023 | Shanghai, China |
| 74 | Caprazol Tablets | Approval No. H20230003 | Jiangsu Kephir Pharmaceuticals Co., Ltd. | 03/08/2023 | Jiangsu, China |
| 75 | Gumetide Tablets | Approval No. H20230005 (Conditional) | Shanghai Haihe Pharmaceuticals Co., Ltd. | 03/07/2023 | Shanghai, China |
| 76 | Leritavir Tablets | Approval No. H20230007 (Conditional) | Guangdong Zhongsheng Reborn Biotech Co., Ltd. | 03/21/2023 | Guangdong, China |
| 77 | Asunaprevir Tablets | Approval No. H20230010 | Nanjing Sanhome Pharmaceuticals Co., Ltd. | 05/12/2023 | Jiangsu, China |
| 78 | Zaberto Monoclonal Antibody Injection | Approval No. S20230028 | Zhejiang Borui Biopharmaceutical Co., Ltd. | 05/12/2023 | Zhejiang, China |
| 79 | Befetimod Tosylate Capsules | Approval No. H20230011 | Betta Pharmaceuticals Co., Ltd. | 05/31/2023 | Zhejiang, China |
| 80 | Shengning Tablets | Approval No. Z20230001 | Guangdong Siji Pharmaceutical Co., Ltd. | 06/08/2023 | Guangdong, China |
| 81 | Voritinib Tablets | Approval No. H20230013 | Betta Pharmaceuticals Co., Ltd. | 06/07/2023 | Zhejiang, China |
| 82 | Annelox Sodium Enteric-Coated Tablets | Approval No. H20230014 | Xianzhu (Beijing) Pharmaceutical Technology Co., Ltd. | 06/21/2023 | Beijing, China |
| 83 | Rimeglitin Tablets | Approval No. H20230017 | Jiangsu Hengrui Medicine Co., Ltd. | 06/27/2023 | Jiangsu, China |
| 84 | Otaconazole Capsules | Approval No. HJ20230084 | eVENUS PHARMACEUTICAL LABORATORIES INC | 06/27/2023 | Canada |
| 85 | Iruar Tablets | Approval No. H20230015 | Qilu Pharmaceuticals Co., Ltd. | 06/27/2023 | Shandong, China |
| 86 | Topafilgrastim Injection | Approval No. S20230036 | Xiamen Amoytop Biotech Co., Ltd. | 06/30/2023 | Fujian, China |
| 87 | Iqosidenib Injection | Approval No. S20230040 (Conditional) | Nanjing Deer BioPharmaceutical Co., Ltd. | 06/30/2023 | Jiangsu, China |
| 88 | Pemotiltide Injection | Approval No. H20230019 | Jiangsu Hansoh Pharmaceutical Group Co., Ltd. | 06/30/2023 | Jiangsu, China |
| 89 | Tolicizumab Injection | Approval No. S20230043 | Innovent Biologics (Suzhou) Co., Ltd. | 08/16/2023 | Jiangsu, China |
| 90 | Shuovitib Tablets | Approval No. H20230023 (Conditional) | Dizal Pharmaceutical Co., Ltd. | 08/22/2023 | Jiangsu, China |
| 91 | Nalsobamab Injection | Approval No. S20230047 (Conditional) | Shanghai Jinmant BioTechnology Co., Ltd. | 09/05/2023 | Shanghai, China |
| 92 | Deuterocidetanib Tablets | Approval No. HJ20230120 | Bristol-Myers Squibb Pharma EEIG | 10/19/2023 | Canada |
| 93 | Litixitinib Tosylate Capsules | Approval No. HJ20230118 | Pfizer Inc. | 10/18/2023 | Germany |
| 94 | Tongluoming Capsules | Approval No. Z20230003 | Yiling Pharmaceutical Co., Ltd. | 10/19/2023 | Hebei, China |
| 95 | Xiaoer Zibei Xuanfei Syrup | Approval No. Z20230002 | Jianmin Pharmaceutical Group Co., Ltd. | 10/19/2023 | Hubei, China |
| 96 | Zhisizong Flavonoid Tablets | Approval No. Z20230004 | Jiangxi Qingfeng Pharmaceutical Co., Ltd. | 10/19/2023 | Jiangxi, China |
| 97 | Epinephrine Injection | Approval No. S20230063 | Wuhan Haite Biopharmaceutical Co., Ltd. | 11/01/2023 | Hubei, China |
| 98 | Gefistotumab Injection | Approval No. SJ20230017 (Conditional) | Roche Pharma (Schweiz) AG | 11/07/2023 | USA |
| 99 | Naxiumab Injection | Approval No. S20230065 (Conditional) | Heyuan Biotech Co., Ltd. | 11/07/2023 | Tianjin, China |
| 100 | Xianglei Tangzuo Ointment | Approval No. ZC20230001 (Conditional) | Uni-President Biotechnology Co., Ltd. | 11/09/2023 | Taiwan |
| 101 | Borritinib Enteric-Coated Capsules | Approval No. H20230027 (Conditional) | Beijing PuRun Pharmaceutical Co., Ltd. | 11/14/2023 | Beijing, China |
| 102 | Aitritavir Tablets/Ritonavir Tablets Combo Pack | Approval No. H20230029 (Conditional) | Fujian Grandpharm Zhonglin Biotechnology Co., Ltd. | 11/23/2023 | Fujian, China |
| 103 | Didasinetib Capsules | Approval No. H20230030 | Zhejiang Jingxin Pharmaceutical Co., Ltd. | 01/02/2024 | Zhejiang, China |

Table S2.

Category 1 innovative drugs approved by NMPA between January and June 2024

| **Drug name** | **Marketing authorization holder** | **Indication** | **Approval date** |
| --- | --- | --- | --- |
| Small Molecule | | | |
| Proline Gagliflozin Tablets | HuiSheng Biopharmaceutical Co., Ltd. | Type 2 diabetes | 01/16/2024 |
| Fumarate Tejigidine Injection | Jiangsu Hengrui Medicine Co., Ltd. | Moderate to severe postoperative abdominal pain | 01/30/2024 |
| Trametinib Capsules | Shanghai Kezhou Pharmaceutical Research Co., Ltd. | Melanoma | 03/12/2024 |
| Iptacopan Hydrochloride Capsules | Novartis Pharma Schweiz AG | Paroxysmal nocturnal hemoglobinuria (PNH) in adults | 04/24/2024 |
| Entinostat Tablets | Taizhou Eirgenix Pharmaceuticals Co., Ltd. | HR-positive, HER2-negative locally advanced or metastatic breast cancer that has recurred or progressed after endocrine therapy | 04/24/2024 |
| Ensartinib Fumarate Capsules | Chia Tai Tianqing Pharmaceutical Group Co., Ltd. | ROS1-positive locally advanced or metastatic non-small cell lung cancer | 04/24/2024 |
| Ripretinib Capsules | Bristol-Myers Squibb Company | ROS1-positive locally advanced or metastatic non-small cell lung cancer | 05/08/2024 |
| Pregabalin Benzenesulfonate Capsules | Haisco Pharmaceutical Group Co., Ltd. | Diabetic peripheral neuropathic pain | 05/15/2024 |
| Rociletinib Methanesulfonate Capsules | Shanghai Bayerda Pharmaceuticals Co., Ltd. | EGFR T790M mutation-positive locally advanced or metastatic non-small cell lung cancer after progression during or after EGFR-TKI therapy | 05/15/2024 |
| Alectinib Citrate Capsules | Chia Tai Tianqing Pharmaceutical Group Co., Ltd. | ALK-positive locally advanced or metastatic non-small cell lung cancer | 06/11/2024 |
| Regorafenib Methanesulfonate Tablets | Nanjing Sanhome Pharmaceuticals Co., Ltd. | EGFR T790M mutation-positive locally advanced or metastatic non-small cell lung cancer | 06/11/2024 |
| Golixetinib Capsules | Dizal (Jiangsu) Pharmaceutical Co., Ltd. | Relapsed or refractory peripheral T-cell lymphoma | 06/18/2024 |
| Cogriptin Tablets | Haisco Pharmaceutical Group Co., Ltd. | Type 2 diabetes | 06/18/2024 |
| Sulbactam Sodium/ Durlobactam Sodium Injection Combo | Entasis Therapeutics, Inc. | Hospital-acquired bacterial pneumonia (HABP) or ventilator-associated bacterial pneumonia (VABP) caused by susceptible strains of Acinetobacter baumannii-calcoaceticus complex in patients 18 years and older | 05/15/2024 |
| Peptide | | | |
| Bempivibatin Citrate Injection | Bio-Thera Solutions Co., Ltd. | Acute coronary syndrome | 06/25/2024 |
| Ecopiclaxone Injection | Novo Nordisk A/S | Type 2 diabetes | 06/18/2024 |
| Monoclonal Antibody | | | |
| Lecanemab Injection | Eisai Inc. | Alzheimer's disease | 01/05/2024 |
| Crovalimab Injection | Roche Pharma (Schweiz) AG | Paroxysmal nocturnal hemoglobinuria (PNH) | 02/06/2024 |
| Bemotuximab Injection | Chia Tai Tianqing Nanjing Shunxin Pharmaceuticals Co., Ltd. | Extensive-stage small cell lung cancer | 04/30/2024 |
| Zimevirumab/Mavoravirumab Injection | Xiamen Biosciences (Suzhou) Co., Ltd. | Passive immune therapy for rabies virus exposure | 06/04/2024 |
| Enlongzumab Injection | CSPC Group Giantstone Biopharmaceutical Co., Ltd. | Recurrent or metastatic cervical cancer expressing PD-L1 after platinum-based chemotherapy failure | 06/25/2024 |
| Bispecific Antibody | | | |
| Ivosidenib Injection | Akeso Biopharma Co., Ltd. | Combined with pemetrexed and carboplatin, for EGFR mutation-positive locally advanced or metastatic non-squamous non-small cell lung cancer that progressed after EGFR-TKI therapy | 05/21/2024 |
| CAR-T | | | |
| Zilokagen Olense Injection | Shanghai CARsgen Therapeutics Co., Ltd. | Relapsed or refractory multiple myeloma | 02/23/2024 |
| Traditional Chinese Medicine | | | |
| Ercha Shangqing Pills | Hubei Qijin Pharmaceuticals Co., Ltd. | Oral ulcers | 01/08/2024 |
| Jiuwei Cough Syrup | Zhuohe Pharmaceuticals Group Co., Ltd. | Cough due to acute tracheobronchitis with wind-heat syndrome (as per Traditional Chinese Medicine) | 02/20/2024 |
| Qinwei Granules | Chengdu Huaxi Natural Pharmaceuticals Co., Ltd. | Acute gouty arthritis | 03/12/2024 |

Table S3.

The first batch of key projects of China's Drug Regulatory Science Action Plan (2019-2021)

| **No.** | **Project title** | **Lead department** | **Implementing units** | **Research focus** |
| --- | --- | --- | --- | --- |
| 1 | Evaluation and Regulatory System for Cell and Gene Therapy Products | Drug Registration Department | Center for Drug Evaluation | Strengthen the evaluation standards and regulatory system for cell and gene therapy, develop guidelines, and promote high-quality development. |
| 2 | Safety Evaluation and Quality Control of Nano Drugs | Drug Registration Department | Center for Drug Evaluation | Research pharmacokinetics, safety evaluation, and quality control of nano drugs, providing guidelines to accelerate approval. |
| 3 | TCM Safety Evaluation Oriented by Clinical Effectiveness | Drug Registration Department, Drug Supervision Department | Pharmacopoeia Commission, Center for Drug Evaluation, National Institutes for Food and Drug Control | Research biological evaluation methods for TCM based on clinical effectiveness and establish quality control systems. |
| 4 | Post-Marketing Drug Safety Monitoring and Evaluation | Drug Supervision Department | Center for Drug Evaluation | Research monitoring methods and real-world data usage, improving post-marketing drug safety evaluation systems. |
| 5 | Evaluation of Drug-Device Combination Products | Medical Device Registration Department, Drug Registration Department | Center for Medical Device Evaluation, Center for Medical Device Standards | Research the evaluation and lifecycle management of drug-device combination products and establish regulatory models. |
| 6 | Safety and Effectiveness of AI Medical Devices | Medical Device Registration Department | Center for Medical Device Evaluation | Research safety and effectiveness evaluation systems for AI medical devices, creating guidelines for regulation. |
| 7 | Regulatory Science for New Materials in Medical Devices | Medical Device Registration Department, Medical Device Supervision Department | Center for Medical Device Evaluation | Research the regulatory mechanisms and risk control of new materials, creating guidelines for quality control and regulation. |
| 8 | Real-World Data in Clinical Evaluation of Medical Devices | Medical Device Registration Department, Medical Device Supervision Department | Center for Medical Device Evaluation | Develop methodologies for using real-world data in medical device evaluation and post-market monitoring. |
| 9 | Safety Evaluation Methods for Cosmetics | Cosmetics Supervision Department | National Institutes for Food and Drug Control | Research safety evaluation methods for cosmetics and develop guidelines for risk assessment and alternative testing. |

Table S4.

The second batch of key projects of China's Drug Regulatory Science Action Plan (2021-2023)

| **No.** | **Project title** | **Lead department** | **Implementing department** | **Research focus** |
| --- | --- | --- | --- | --- |
| 1 | Research on Efficacy, Safety, and Quality Control of Traditional Chinese Medicine (TCM) | Drug Registration Department | Center for Drug Evaluation, National Institutes for Food and Drug Control, Center for Food and Drug Inspection, Pharmacopoeia Commission | Develop tools and standards for TCM efficacy, safety (toxicity), and quality control across production processes. |
| 2 | Evaluation System for Stem Cell and Gene Therapy Products | Drug Registration Department, Drug Supervision Department | Center for Drug Evaluation, National Institutes for Food and Drug Control, Center for Food and Drug Inspection | Create evaluation methods for stem cell and gene therapy products to enhance regulatory approval processes. |
| 3 | Real-World Data for Evaluating TCM, Rare Disease Drugs, and Innovative Medical Devices | Drug Registration Department, Medical Device Registration Department | Drug and Medical Device Evaluation Centers, Center for Food and Drug Inspection | Develop standards and tools for using real-world data to support evaluations of TCM, rare disease drugs, and devices. |
| 4 | Evaluation of Diagnostic and Therapeutic Products for Emerging Infectious Diseases | Drug Registration Department, Medical Device Registration Department | National Institutes for Food and Drug Control, Drug and Medical Device Evaluation Centers | Research diagnostics and treatments for emerging infectious diseases, establishing evaluation standards and guidelines. |
| 5 | Evaluation of Nano-Based Innovative Drugs and Medical Devices | Drug Registration Department, Medical Device Registration Department | Drug and Medical Device Evaluation Centers, National Institutes for Food and Drug Control, Center for Food and Drug Inspection | Study safety, efficacy, and quality control for nano-based drugs and devices, and create evaluation standards. |
| 6 | Evaluation of Innovative Medical Devices (Remote Transmission, Flexible Electronics, Medical Robots) | Medical Device Registration Department | Medical Device Evaluation Center, National Institutes for Food and Drug Control, Center for Food and Drug Inspection | Assess innovative medical devices like remote transmission tech, wearables, and medical robots, and develop guidelines. |
| 7 | Evaluation of New Biomaterials | Medical Device Registration Department, Cosmetics Supervision Department | Medical Device Evaluation Center, National Institutes for Food and Drug Control, Center for Food and Drug Inspection | Research new biomaterials like 3D printing, tissue-engineered products, and antimicrobial materials, setting evaluation standards. |
| 8 | Guidelines for New Cosmetic Ingredients and Safety Monitoring | Cosmetics Supervision Department | National Institutes for Food and Drug Control, Drug Evaluation Center, Center for Food and Drug Inspection | Develop standards and monitoring methods for new cosmetic ingredients and safety evaluations. |
| 9 | Evaluation Tools and Standards for Common Diseases and Cancer | Drug Registration Department, Medical Device Registration Department | Drug and Medical Device Evaluation Centers, National Institutes for Food and Drug Control, Center for Food and Drug Inspection | Create evaluation tools and standards for diagnostic and therapeutic products for cancer and other common diseases. |
| 10 | Research on Vigilance Technologies for Drugs and Medical Devices | Drug Supervision Department, Medical Device Supervision Department | Drug and Medical Device Evaluation Centers, National Institutes for Food and Drug Control, Center for Food and Drug Inspection | Strengthen vigilance technologies for monitoring drug and medical device safety and building smarter reporting systems. |
